# How to improve the dissemination of clinical practice guidelines in the Brazilian Unified Health System? Report of a pilot project

**DOI:** 10.1186/s12961-023-00966-y

**Published:** 2023-03-23

**Authors:** Viviane Cássia Pereira, Sarah Nascimento Silva, Ávila Teixeira Vidal, Gustavo Veiga e Lins, Jorge Otávio Maia Barreto

**Affiliations:** 1 Oswaldo Cruz Foundation, Brasília, Brazil; 2 Brazilian Ministry of Health, Brasília, Brazil

**Keywords:** Guidelines, Guideline implementation, Public health system

## Abstract

In Brazil, there have been some initiatives to improve the development of Ministry of Health clinical protocols and therapeutic guidelines (PCDTs in Portuguese, and clinical practice guidelines—CPGs, in English) and their implementation so that best practices can be disseminated and adopted at multiple levels of health systems. One of the initiatives was to conduct a pilot project to improve the format of these CPGs. The objective of this article is to present the processes and results of the pilot project, including the development of a new standardized format for CPGs to promote national dissemination and uptake. The pilot project was designed in three phases: identification and selection of strategies to effectively implement clinical practice guidelines, definition of the ideal characteristics for the format of CPGs, and development and implementation of the new format. Initially, an overview of systematic reviews was conducted to map the global evidence on the effectiveness of dissemination and implementation strategies of CPGs. Among the most effective interventions, a low-cost strategy was selected to improve the format of CPGs, namely a full format and a short format. The two formats were evaluated for usefulness and acceptability by professionals who use or develop CPGs, and after several reiterations, the formats were finalized, considering the progression of care (from diagnosis of the disease to treatment, including specific technologies indicated in each stage of the disease). Related to the technical aspects, the visual presentation of the CPGs was improved, ensuring that key information was easily identified for decision-making by end users. The initial phase of implementation involved 33 clinical conditions, equating to approximately 20% of published CPGs. It is anticipated that disseminating the CPGs in the new formats will promote the accessibility of information and implementation of standardized CPGs by health professionals in the public health sector (servicing more than 210 million Brazilians). Further research should be considered to determine the impact of the use of the new CPGs formats, contributing to the knowledge base related to the implementation of guidelines in Brazil and internationally.

**Supplementary Information:**

The online version contains supplementary material available at 10.1186/s12961-023-00966-y.

## Background

Clinical practice guidelines (CPGs) are systematically developed statements with recommendations based on systematic reviews to aid clinician and patient decision-making regarding the benefits and harms of care options [1]. CPGs provide standardized guidance for healthcare and may impact the quality of care and impact individual and collective public health outcomes [2].

In Brazil, CPGs or National Guidelines are called “clinical protocols and therapeutic guidelines” (*protocolos clínicos e diretrizes terapêuticas—*PCDTs, in Portuguese, from now on referred to as CPGs) [3]. CPGs “establish criteria for the diagnosis of diseases or health problems; the recommended treatment, with medications and other appropriate products, when appropriate; the recommended dosages; the mechanisms of clinical control; and the monitoring and verification of therapeutic results, to be followed by the Brazilian Unified Health System (*Sistema Único de Saúde*—SUS, in Portuguese) managers.” Currently, CPGs guide more than 160 clinical condition CPGs, guiding clinical practice within the SUS [4].

The development of CPGs at the national level is the responsibility of the Brazilian Ministry of Health, assisted by the National Commission for the Incorporation of Technologies in the SUS (CONITEC, acronym in Portuguese) [5]. Adaptation of published good-quality guidelines that use the Grading of Recommendations, Assessment, Development and Evaluation (GRADE) approach, to the local Brazilian context, has assisted the Ministry in efficiently producing CPGs that are of improved quality. This ensures the adoption of best practices within the public health system [6, 7].

The implementation of CPGs has been widely discussed in the global scientific literature. Several studies address the factors that impact this process, especially identifying the barriers and facilitators to determine the changes required in specific contexts [8–10]. In addition, the format and clarity of CPGs can influence the successful implementation of the included recommendations [11].

Therefore, a more structured presentation of the information, including formatting, summarizing the key recommendations, providing the strength of the recommendations and the quality of the evidence, and using different versions of a CPG, targeting healthcare workers and the public, may be potential options to improve the implementation and adherence of guidelines [12, 13].

One of the initiatives developed by the Executive Secretariat of CONITEC in partnership with the Osvaldo Cruz Foundation (Fiocruz, Brasília) is the project “Support for the improvement of technology management in the SUS through a platform for the translation, exchange and social appropriation of knowledge”. The project aims to improve the development and implementation of CPGs in the national health system, including training of health technicians and managers (CPG preparation course) and developing and implementing an improved format proposal for CPGs [14].

This article presents the processes and results of the pilot project for developing the best format for CPGs released by the Brazilian Ministry of Health for nationwide implementation, in a standardized manner.

## Phases of development of the pilot project

**Fig. 1:**
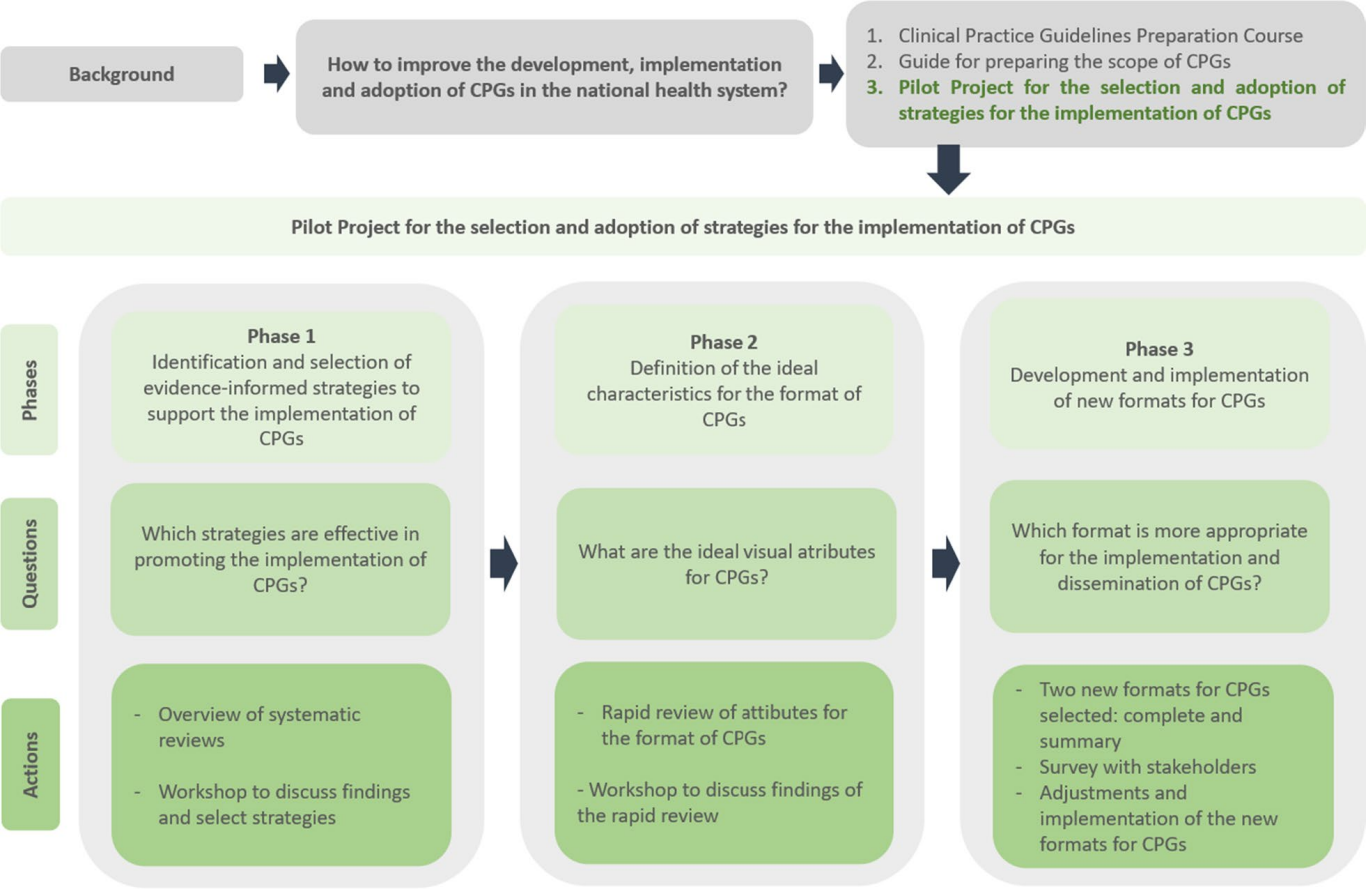
Phases of the pilot project for selecting and adopting a strategy for implementing and disseminating CPGs (Source: own elaboration)

Fiocruz Brasília implemented a pilot project “Support for the improvement of technology management in the SUS through a platform for the translation, exchange, and social appropriation of knowledge” between 2016 and 2022. Commissioned and funded by the Secretary of Science, Technology, Innovation and Inputs Strategic Supplies of the Brazilian Ministry of Health, this project was developed in close collaboration with the Department of Management and Incorporation of Technologies and Innovation in Health. The three phases of the pilot project included (1) identification and selection of evidence-informed strategies to support the implementation of CPGs; (2) definition of the ideal characteristics for the format of CPGs; and (3) development and implementation of new formats for CPGs. Each phase was guided by questions to determine the respective action(s), as shown in Fig. 1.

### Step 1—selection of strategies to support the implementation of PCDTs

#### Overview of systematic reviews

The first phase focused on answering the following questions: (i) Which strategies are effective in supporting the implementation of clinical guidelines? (ii) Which of these strategies are more appropriate for the SUS context? For the first question, an overview of systematic reviews was conducted to map global evidence for effective strategies to disseminate and implement CPGs. Details of the overview can be found in the full report [15].

Educational materials, educational meetings (training), reminders and auditing with feedback appeared to be the most effective interventions, suggesting that these strategies could be offered as first choices in a guideline implementation process [15].

#### Workshop to discuss findings and select a CPG implementation strategy

Based on the results of the overview, a workshop was conducted for representatives of the institutions involved in the project to identify the most appropriate CPG implementation strategies. Researchers from Fiocruz Brasília and technicians from the Ministry of Health who participated in the Technical Subcommittee on CPG Evaluation of CONITEC participated in this workshop in 2018.

Three potential strategies were identified: (1) educational materials, (2) educational meetings and (3) reminders. These strategies were considered to be low-cost, flexible and simpler compared to other strategies and were proposed to be used synergistically. However, the initial and most feasible priority was to improve the format of CPGs for ease of use by the end user and to encourage implementation.

### Step 2—definition of the ideal characteristics for the format of PCDTs

#### Rapid review

To guide the reformatting of CPGs, a rapid review was conducted on the visual presentation of CPGs that could potentially improve effective use and implementation. The rapid review was conducted to answer the following question: What are the main domains and attributes related to the format of clinical guidelines that favour dissemination and use?

Published studies were retrieved from a search conducted in three indexed databases (without language or date restrictions) according to predefined criteria. Of the 129 articles that were retrieved, 14 were read in full text, and six were included in the rapid review. The data from the selected studies were extracted and summarized in an evidence table (including study year, authors, title, objective, country, domains, subdomains, attributes, definition of attributes related to format and message, summary of evidence by attribute, barriers and facilitators, instrument to evaluate capture/usability, and source of funding) (Additional file 1) [16–21].

#### Communication of content: the most appropriate format

Developing the most feasible format for CPGs comprises a two-step process: (1) the creation of content and (2) the effective communication of the content (message and format), the latter being the focus of the rapid review [16]. The studies selected in the review suggested that the domain “format” can be subdivided into six subdomains: (i) multiple versions of the guidelines, (ii) forms of delivery, (iii) document components, (iv) structure and organization of the text, (v) document layout and (vi) presentation of textual and non-textual information. For each sub-domain, the main characteristics were collected, and respective recommendations were established based on the identified evidence to contribute to greater utility and acceptance of the clinical guidelines (Additional file 2).

#### Workshop to discuss the findings

To clearly describe the format to be adopted in CPGs published by the Ministry of Health, a second workshop was conducted with the same researchers as the previous workshop. The attributes that were considered priorities were defined and adopted to guide a proposal to reformat the CPGs released by the Ministry of Health. Table 1 summarizes the results of this discussion.

**Table 1:** Main attributes for redesigning the format of CPGs

Subdomain	Main attributes
Multiple versions of the guidelines	End users: health professionals
	Document types:
	(1) Layout/format for the full protocol (dynamic/static)
	(2) Layout/summary format for the protocol (static)
	(3) Interactive algorithm, with field filling options (static/dynamic)
Form of delivery	Available on the CONITEC website in PDF version for download
Components of the document (subject to variation depending on the topic)	1. Introduction
	2. International Statistical Classification of Diseases and Related Health Problems (ICD-10)
	3. Diagnosis
	3.1 Clinical diagnosis
	3.2 Laboratory diagnosis
	3.3 Diagnosis by imaging examinations
	3.4 Other exams
	4. Inclusion criteria
	5. Exclusion criteria
	6. Special cases
	7. Reference centre
	8. Treatment
	8.1 Non-drug treatment
	8.2 Drug treatment
	8.3 Drugs
	8.4 Management schemes
	8.5 Treatment time–interruption criteria
	8.6 Expected benefits
	9. Monitoring
	10. Post-treatment follow-up
	11. Regulation/control/evaluation by the manager
	12. Statement of clarification and responsibility
	13. Bibliographic references
	Appendix 1 Literature search and evaluation methodology
Presentation: structure and organization of the text; layout	CPG (full version):
	– Sources used: Calibri and Montserrat
	– Font size: 25 pt for titles; 13 pt for body; 9 pt for tables; 8 pt for footers; and 20 pt for the cover title
	– Colours used: four types of CPGs were diagrammed to identify the type of protocol (blue, green, yellow and purple)
	– Cover page with an image and graphic elements
	– Text formatted in two columns in the main body and one column in the annexes and appendixes
	– Structure—cover page, table of contents, summary, main content, references, appendix, annex and back cover
	– Tables and images with one column, regardless of location
	– Large tables presented in horizontal format
	– Internal graphic elements include the page number, name of the book, signature of the Ministry of Health and indicator for the end of each page
	– Text spacing configured for comfortable reading, with 15-pt line spacing
	– Titles break the sequence of two columns of paragraphs to facilitate the understanding of the reader
	– Tables with alternating row colours to facilitate visualization
	– Figure entries for the annex and appendix, with large font and the image in the background to break the text
	– Back cover with graphic elements, insignia of the Ministry of Health, address of the virtual library of the Ministry of Health and the International Standard Book Number
	CPG (short version):
	– Maximum of four pages preferably
	– Elements of the cover page placed at the top of the first page to reduce the number of pages
	– A “summary CPG” logo placed at the top of each new document
	– Iconography created for each type of CPG in rounded blue colour with the icon in dark blue
	– Colours used: four types of CPGs were diagrammed to identify the type of protocol (blue, green, yellow and purple)
	– Font size: 13 pt for titles; 9 pt for the body; 8 pt for tables; 6 pt for footers; and 20 pt for the title
	– Sources used: Calibri and Montserrat
	– Several templates developed for short CPGs so that various styles of text and elements are available for use

Source: the authors

### Step 3—development and implementation of new formats for CPGs

#### Development of new formats for CPG

Two new formats were developed for CPGs released by the Brazilian Ministry of Health. One format included the comprehensive guideline (CPG—full version), and the other, a summarized version of the content (CPG—short version). Both formats were developed through the collaboration of researchers from Fiocruz Brasília, technicians from the Ministry of Health and a professional designer. The process of preparing the layout for the full format considered a logical description of the care pathway (from the definition and diagnosis of the disease to the management of the disease, using various technologies). Further to the technical aspects, visual presentations were included for ease of use by end users. These formats were generated in accordance with the editorial standards established by the Ministry of Health Editor, to be indexed and included in the Virtual Health Library database (indexed database for consultation in the health area in the Americas) [22].

The shorter or summarized version of the CPG focused on key strategic information as indicated by the technicians within the Ministry of Health that are responsible for preparing CPGs. This format provides a quick reference and tool for health professionals in the Brazilian public health system to diagnose and manage clinical conditions. Visual presentation of relevant sections (including content on evidence syntheses), according to the required standards, was considered to promote ease of understanding, greater usability and uptake by the end user.

The decision to adopt two new dissemination formats, a comprehensive and a summary version, was informed by the strategies identified in the rapid review, guidance by the Ministry of Health technicians who prepare these documents and the public, who regularly use the documents. The development process considered best practices for presenting digital books to facilitate access to information, management and the understanding of content by users. Generic structured models were developed to facilitate standardized development using the same visual identity going forward. The layout was configured with adequate spacing between lines and between letters, ideal font sizes, indicative colours and adequate space between elements for comfortable reading by the end user. The shorter summarized version was configured to a synthesized format, with the greatest possible clarity, inspired by infographics, a space-saving design, iconography and indicative graphic elements. Thus, in addition to comfort when reading, the user can recognize the identity of products in different versions.

#### Survey with stakeholders

A survey was conducted by the Ministry of Health, according to the terms of Resolution No. 510, of 7 April 2016, to choose the final standards to be adopted. No ethics approval was required. The survey followed a questionnaire format that captured stakeholders’ opinions on the usefulness and acceptability of the new formats that were proposed in the pilot project. The most interesting information and layout elements from the users’ perspective were identified to inform the final summary format. The research (in the form of a questionnaire) was conducted during the first congress of the Brazilian Health Technology Assessment Network (*Rede Brasileira de Avaliação de Tecnologias em Saúde*—REBRATS, in Portuguese) held in October 2019 amongst professionals who use or develop CPGs in their practice [23].

Survey questions included profiling respondents and aspects related to the use and preferences of formats for CPGs and requested participants to choose one of the three formats. The presented layouts had the same basic colours and similar general layouts, varying in some elements, such as the presentation of content and the distribution of information. In the questionnaire, the participants also had an opportunity to provide his/her opinion of the new format, the structure of the model, the key sections that should be included in the short version and suggestions regarding the size of the document. A total of 154 people responded to the questionnaire, from all regions of Brazil. More than 50% of the participants reported that they consult Brazilian CPGs weekly; with 19.1% reporting biweekly or monthly use; and only 2.2% not knowing or reporting rare consultations. The short version model with the most votes featured treatment flow diagrams and algorithms (68.2%). Participants had a positive initial experience with the summarized format, with most highlighting the importance of this format for the dissemination of Brazilian CPGs. Regarding the attributes of the formats presented, most indicated that they had a coherent structure and were useful for professional practice, with an appropriate and pleasing colour scheme. Most preferred a shorter document of one or two pages (61.7%). General comments on the models were positive, emphasizing the inclusion of flow diagrams and tables, without making the document too busy with excessive images and visual information. This input was important, and the final format of the summarized, shorter version of the CPG was amended accordingly.

The proposed summarized format of the CPG was peer-reviewed by the technicians who work specifically on the preparation and updating of these documents in the Ministry of Health. The main adjustments were inserting figures, flow diagrams and sources (letters) and strictly following the official editorial guidelines. Visual amendments included text alignment for ease of reading and standardized presentation of sections and titles to facilitate easier identification by readers.

#### Adjustments to layouts and the implementation of new CPG formats

**Fig. 2:**
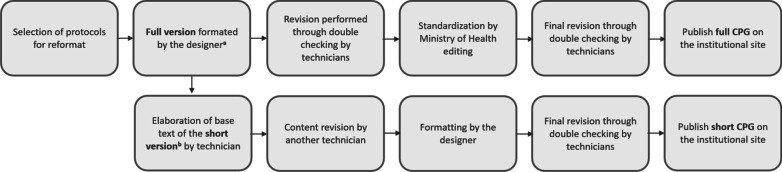
Process flow of format preparation, review and approval. Source: the authors^a^Full version: format in which the information is detailed, with all the key recommendations, point-of-care decisions and the rationale for decision-making, including the literature search and evaluation methodology at the end. ^b^Short version: format in which the information is the summarized for point-of-care decisions, with self-explanatory figures, flows and reduced text

To provide functional adjustments to the layouts and the guideline content, a technical team composed of five technicians from the Executive Secretariat of CONITEC was appointed. A process flow was established to outline the steps and due dates for the reiterative review and of the CPGs until they were approved for publication (Fig. 2).

For the comprehensive PCDT (PCDT—full version), the technical team adapted parameters defined by the Ministry of Health publisher and amended the narrative to align with the adopted format and layout ascribed by the designer.

Multiple steps were involved in developing the summarized version, undertaken by dedicated experienced technicians. The designer utilized diagrams, tables and explanatory figures to identify relevant information in each section of the document. Technical peer review of most documents ensued, whilst some complex topics required extended team meeting discussions with input from professionals with different expertise to decide on the final format. Collaboration between healthcare professionals and designers created opportunities to explore the translation of knowledge, transforming extremely technical and complex documents into summarized versions that would be easy to understand for any reader. For the initial implementation of the new formats, CPGs already published by the Ministry of Health were selected and updated to the new versions (full and short). Other factors that were considered were the frequency of confusion amongst professionals, the complexity of the content and the time of publication of the documents. The CPGs that were included in the initial stage of implementation of the new layout are available on the CONITEC website (http://conitec.gov.br/index.php/protocolos-e-diretrizes) and in the examples presented in Additional files 3 and 4.

## Discussion

This article described a structured institutional process to identify strategies to improve the dissemination and implementation of PCDTs released by the Brazilian Ministry of Health. The various phases were established on the best available evidence with extensive collaboration between the research team and the Ministry of Health. A review of systematic reviews of the evidence identified several strategies, but low-cost, flexible and simple initiatives such as educational materials and reminders, implemented synergistically, were preferred. However, through consensus it was determined that the initial and most feasible priority was to improve the format of PCDTs for ease of use by the end user and to encourage implementation—and thus, a comprehensive format and a summarized version with relevant visual presentations were developed. This pilot project suggested that poor uptake of CPGs is possibly related to issues with the disseminated material, resulting in inadequate communication of the required message(s).

The definition of the ideal characteristics for the CPGs’ formats was informed by evidence (rapid review) and by the stakeholders’ opinions on the usefulness and acceptability of the new formats—and thus, a comprehensive version and a summarized version with relevant visual presentations were developed. The attributes for redesigning the CPGs are aligned with the recent and validated Guideline Language and Format Instrument (GLAFI), which presents the importance of the subdomains we used for recommendation uptake [24].

The new versions of CPGs were implemented, and the next step is evaluating the effectiveness and acceptability amongst the end users to identify the main barriers for implementation and to the understanding of the factors that influence guideline acceptance and adoption. Seeking the improvement of the CPGs implementation, the Ministry of Health requested interactive algorithms and other options of dynamic formats, but they are still in development. The use of these electronic formats for guidelines may impact the constructs configurations used in the pilot project.

A Cochrane systematic review by Baker et al. [25] concluded that strategies adapted to address barriers can further improve professional practice when compared with no intervention or the simple dissemination of guidelines. In Brazil, a similar project was conducted to identify barriers and strategies for the implementation of the National Childbirth Guidelines. The identification of barriers was followed by a deliberative dialogue about the respective interventions to overcome these barriers. The following interventions were selected: to promote the use of multifaceted interventions, and educational interventions, to conduct auditing and provide feedback to change professional practice, to provide reminders, to permit patient-mediated interventions, and engaging decision-makers to promote the use of guidelines [26].

Facing the need to quickly update evidence and disseminate it, some initiatives have been developed, mainly driven by the COVID-19 pandemic. An example of this is the MAGICapp platform, a web-based collaborative tool for developed, published and dynamically updated, trustworthy and living guidelines. According to the platform, for uptake improvement, usability and comprehensibility, guidelines need to be created in electronic presentation formats, allowing large content adaptation and update at different levels of detail (multilayered presentation) [27].

Our study has limitations. Even though the overview of the systematic reviews on CPGs’ implementation strategies have been conducted with rigorous methodology—the rapid review on CPG formats adopted methodological shortcuts, and, therefore, relevant scientific evidence may not have been selected. Another important limitation was the fact that the implementation of the new formats consisted in publishing the guidelines on a web page, without active implementation directed audience targeting. Lastly, the ideal attributes for evaluating the language used in the guidelines as part of the “communicating” content (language and format), one of the main determinants of implementation for optimal uptake, were not addressed.

## Conclusion

Comprehensive guidelines and guideline summaries, with restructured layouts, are freely accessible and are available in the Virtual Health Library database databases for the Americas, and from the Ministry of Health portal.

In conclusion, it is anticipated that the updated formats adopted by the Ministry of Health will impact the dissemination of public health sector CPGs and improve access to information by healthcare professionals for the provision of standardized healthcare for the Brazilian population. Further research is needed to determine the impact of the new CPG formats, which will further contribute to the knowledge base related to the implementation of guidelines in Brazil and globally.

## Supplementary Information

**Additional file 1: Table S1.** Literature search, selection and characteristics of the studies.

**Additional file 2: Table S2.** Subdomains and attributes used in the standardization of the format for clinical guidelines.

**Additional file 3.** Example of a CPG, full version.

**Additional file 4.** Example of a CPG, short version.

## Data Availability

The datasets used and/or analysed during the current study are available from the corresponding author upon reasonable request.

## Abbreviations

CONITEC: National Commission for the Incorporation of Technologies in the Brazilian Unified Health System
CPG: Clinical practice guidelines
Fiocruz: Osvaldo Cruz Foundation
PCDT: Clinical protocols and therapeutic guidelines
REBRATS: Brazilian Health Technology Assessment Network
SUS: Brazilian Unified Health System

## References

[1] Institute of Medicine (US) Committee on Standards for Developing Trustworthy Clinical Practice Guidelines. Clinical practice guidelines we can trust. In: Graham R, Mancher M, Wolman DM, Greenfield S, Steinberg E, editors. Clinical practice guidelines we can trust. Washington (DC): National Academies Press (US); 2011. 24983061

[2] Wieringa S, Dreesens D, Forland F, Hulshof C, Lukersmith S, Macbeth F, Shaw B, van Vliet A, Zuiderent-Jerak T, AID Knowledge Working Group of the Guidelines International Network (2018). Different knowledge, different styles of reasoning: a challenge for guideline development. BMJ Evid Based Med.

[3] Brasil. Ministério da Saúde. Comissão Nacional de Incorporação de Tecnologias no Sistema Único de Saúde. Protocolos e Diretrizes. http://conitec.gov.br/protocolos-e-diretrizes. Accessed 25 Oct 2021.

[4] Brasil. Ministério da Saúde. Comissão Nacional de Incorporação de Tecnologias no Sistema Único de Saúde. PCDT em elaboração. 2019. http://conitec.gov.br/pcdt-em-elaboracao. Accessed 2 Sept 2021.

[5] Brasil. Lei no 12.401, de 28 de abril de 2011. Altera a Lei no 8.080, de 19 de setembro de 1990, para dispor sobre a assistência terapêutica e a incorporação de tecnologia em saúde no âmbito do Sistema Único de Saúde - SUS. In: Diário Oficial da União. 2011. p. 1–2. http://www.planalto.gov.br/ccivil_03/_ato2011-2014/2011/lei/l12401.htm. Accessed 14 Mar 2018.

[6] Brasil. Ministério da Saúde. SCTIE. Guia de elaboração de protocolos clínicos e diretrizes terapêuticas: delimitação do escopo.. Brasília: Ministério da Saúde. 2019. https://bvsms.saude.gov.br/bvs/publicacoes/guia_elaboracao_protocolos_delimitacao_escopo_2ed.pdf. Accessed 2 Sep 2021.

[7] Brasil. Ministério da Saúde. Secretaria de Ciência, Tecnologia e Insumos Estratégicos. Departamento de Gestão e Incorporação de Tecnologias em Saúde. Diretrizes metodológicas: elaboração de diretrizes clínicas. Brasília: Ministério da Saúde. 2016. https://bvsms.saude.gov.br/bvs/publicacoes/diretrizes_metodologicas_elaboracao_diretrizes_metodologicas.pdf.

[8] Grimshaw JM, Thomas RE, MacLennan G, Fraser C, Ramsay CR, Vale L, Whitty P, Eccles MP, Matowe L, Shirran L, Wensing M, Dijkstra R, Donaldson C. Effectiveness and efficiency of guideline dissemination and implementation strategies. Health Technol Assess. 2004;8(6):iii–iv, 1–72. 10.3310/hta8060. 10.3310/hta8060 14960256

[9] Hakkennes S, Dodd K (2008). Guideline implementation in allied health professions: a systematic review of the literature. Qual Saf Health Care.

[10] Krause J, Van Lieshout J, Klomp R, Huntink E, Aakhus E, Flottorp S, Jaeger C, Steinhaeuser J, Godycki-Cwirko M, Kowalczyk A, Agarwal S, Wensing M, Baker R (2014). Identifying determinants of care for tailoring implementation in chronic diseases: an evaluation of different methods. Implement Sci.

[11] Gagliardi AR, Brouwers MC, Palda VA, Lemieux-Charles L, Grimshaw JM (2011). How can we improve guideline use? A conceptual framework of implementability. Implement Sci.

[12] Yang N, Yu Y, Zhang A, Estill J, Wang X, Zheng M, Zhou Q, Zhang J, Luo X, Qian C, Mao Y, Wang Q, Yang Y, Chen Y (2019). Reporting, presentation and wording of recommendations in clinical practice guideline for gout: a systematic analysis. BMJ Open.

[13] Schünemann HJ, Wiercioch W, Etxeandia I, Falavigna M, Santesso N, Mustafa R, Ventresca M, Brignardello-Petersen R, Laisaar KT, Kowalski S, Baldeh T, Zhang Y, Raid U, Neumann I, Norris SL, Thornton J, Harbour R, Treweek S, Guyatt G, Alonso-Coello P, Reinap M, Brozek J, Oxman A, Akl EA (2014). Guidelines 2.0: systematic development of a comprehensive checklist for a successful guideline enterprise. CMAJ.

[14] Brasil. Fundação Oswaldo Cruz. Guias de implementação iGUIAS | Apoio ao aprimoramento da gestão de tecnologias no SUS. Fiocruz. https://brasilia.fiocruz.br/aagts/guias-de-implementacao-iguias/. Accessed 28 Oct 2021.

[15] Pereira VC, Silva SN, Carvalho VKS, Zanghelini F, Barreto JOM (2022). Strategies for the implementation of clinical practice guidelines in public health: an overview of systematic reviews. Health Res Policy Syst.

[16] Kastner M, Makarski J, Hayden L, Durocher L, Chatterjee A, Brouwers M, Bhattacharyya O (2013). Making sense of complex data: a mapping process for analyzing findings of a realist review on guideline implementability. BMC Med Res Methodol.

[17] Kastner M, Estey E, Hayden L, Chatterjee A, Grudniewicz A, Graham ID, Bhattacharyya O (2014). The development of a guideline implementability tool (GUIDE-IT): a qualitative study of family physician perspectives. BMC Fam Pract.

[18] Kastner M, Bhattacharyya O, Hayden L, Makarski J, Estey E, Durocher L, Chatterjee A, Perrier L, Graham ID, Straus SE, Zwarenstein M, Brouwers M (2015). Guideline uptake is influenced by six implementability domains for creating and communicating guidelines: a realist review. J Clin Epidemiol.

[19] Grudniewicz A, Bhattacharyya O, McKibbon KA, Straus SE (2016). User-centered design and printed educational materials: a focus group study of primary care physician preferences. J Contin Educ Health Prof.

[20] Grudniewicz A, Bhattacharyya O, McKibbon KA, Straus SE (2015). Redesigning printed educational materials for primary care physicians: design improvements increase usability. Implement Sci.

[21] Gupta S, Rai N, Bhattacharrya O, Cheng AYY, Connelly KA, Boulet LP, Kaplan A, Brouwers MC, Kastner M (2016). Optimizing the language and format of guidelines to improve guideline uptake. CMAJ.

[22] Brasil. Ministério da Saúde. Secretaria-Executiva. Subsecretaria de Assuntos Administrativos. Procedimentos para Normalização de Publicações do Ministério da Saúde. Brasília, DF; 2018. https://bvsms.saude.gov.br/bvs/publicacoes/procedimentos_normalizacao_publicacoes_ministerio_saude_2ed.pdf. Accessed 28 Oct 2021.

[23] REBRATS 2019 - I CONGRESSO DA REBRATS. 2019. https://eventos.galoa.com.br/rebrats-2019/page/544. Accessed 26 Oct 2021.

[24] Gupta S, Tang R, Petricca K, Florez ID, Kastner M (2022). The guideline language and format instrument (GLAFI): development process and international needs assessment survey. Implement Sci.

[25] Baker R, Camosso-Stefinovic J, Gillies C, Shaw EJ, Cheater F, Flottorp S, Robertson N, Wensing M, Fiander M, Eccles MP, Godycki-Cwirko M, van Lieshout J, Jäger C (2015). Tailored interventions to address determinants of practice. Cochrane Database Syst Rev.

[26] Barreto JOM, Bortoli MC, Luquine CD, Oliveira CF, Toma TS, Ribeiro AAV, Tesser TR, Rattner D, Vidal A, Mendes Y, Carvalho V, Neri MA, Chapman E (2020). Implementation of national childbirth guidelines in Brazil: barriers and strategies. Rev Panam Salud Publica.

[27] MAGIC. Trustworthy guidelines, evidence summaries and decision aids that we can all use and share. MAGIC Foundation. https://magicevidence.org/. Accessed 20 Nov 2022.

[28] Brasil. Ministério da Saúde. Conselho Nacional de Saúde. Resolução no 510, de 7 de abril de 2016. Trata sobre as diretrizes e normas regulamentadoras de pesquisa em ciências humanas e sociais. Diário Oficial da União, Brasília, DF, 24 maio 2016. https://www.in.gov.br/materia/-/asset_publisher/Kujrw0TZC2Mb/content/id/22917581.

