# Supplementary material for: How to improve the dissemination of clinical practice guidelines in the Brazilian Unified Health System? Report of a pilot project
Source: Health Res Policy Syst. 2023 Mar 23;21:22. doi: 10.1186/s12961-023-00966-y (PMC10035473; doi:10.1186/s12961-023-00966-y)
Supplement: Supplementary file 1 — Additional file 1: Table S1. Literature search, selection and characteristics of the studies. [file 12961_2023_966_MOESM1_ESM.docx]

Additional file 1 - Literature search, selection and characteristics of the studies

Table S1 - Criteria for study selection

| **Category** | **Inclusion criteria** |
| --- | --- |
| Documents | Clinical guidelines |
| Intervention | Attributes related to the format/design of clinical guidelines |
| Comparator | No restriction |
| Outcomes | Capture/acceptance and use of the document by health professionals |
| Study design | No restriction |

Source: the authors

Table S2 - Search strategy

| PUBMED (NCBI) until 08/29/2019 | | |
| --- | --- | --- |
| #1 | ("educational material" [Title] OR "educational materials" [Title] OR guideline [Title]) | 13.964 |
| #2 | (uptake OR usability) | 379.774 |
| #3 | (format OR design) | 1.480.653 |
| #4 | #1 AND #2 AND #3 | 41 |
| Cochrane Library until 08/29/2019 | | |
| #1 | (educational material): ti OR (educational materials): ti OR (guideline): ti | 2700 |
| #2 | (uptake OR usability): ti, ab, kw AND (format OR design): ti, ab, kw | 5375 |
| #3 | #1 AND #2 | 26 |
| EMBASE until 08/29/2019 | | |
| #1 | 'educational material': ti OR 'educational materials': ti OR guideline: ti | 19.038 |
| #2 | format OR design | 1.605.048 |
| #3 | uptake OR usability | 509,396 |
| #4 | #1 AND #2 AND #3 | 62 |

Source: the authors

Figure S1 - Flowchart and selection of studies on attributes for the format and layout of clinical guidelines


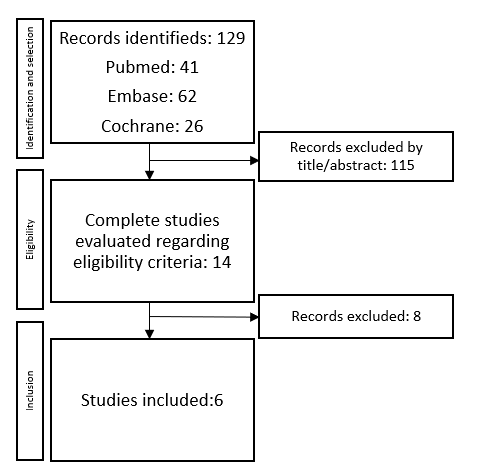


Source: the authors

Table S3 - Characteristics of the included studies

| Author, year | Title | Design | Country | Objectives |
| --- | --- | --- | --- | --- |
| Kastner 2013 | Making sense of complex data: a mapping process for analyzing findings of a realist review of guideline implementability | Realistic review | Canada | Describe the development of an analytical process to organize and synthesize data from a realistic review |
| Kastner 2014 | The development of a guideline implementability tool (GUIDE-IT): a qualitative study of family physician perspectives | Qualitative study - semi -structured interview | Canada | Explore how family physicians perceive the implementability of guidelines and to determine which components should be included in the final prototype of the Guidelines Implementability Tool (GUIDE-IT) |
| Kastner 2015 | Guideline uptake is influenced by six implementability domains for creating and communicating guidelines: a realist review | Realistic review | Canada | Identify factors associated with the implementation of clinical practice guidelines and recommendations through a comprehensive and multidisciplinary perspective and determine which characteristics can improve the acceptance of guidelines |
| Grudniewicz 2015 | Design redesign in educational materials for primary care physicians: design improvements increase usability | Qualitative study - discrete choice experiment | Canada | Evaluate how printed educational materials can be redesigned to better meet the needs of primary care physicians and whether usability and selection can be improved when design principles and user preferences are used |
| Grudniewicz 2016 | User-centered design and printed educational materials: A focus group study of primary care physician preferences | Qualitative study - focus groups | Canada | Collect the preferences of primary care physicians for the design of and content in printed educational materials oriented toward physicians and determine the main attributes that can increase their usability and acceptance |
| Gupta 2016 | Optimizing the language and format of guidelines to improve guideline uptake | Narrative review | Canada | Explore how the language and format used in clinical guidelines can affect the likelihood that they will be used in clinical practice |

Source: the authors
