# Supplementary material for: How to improve the dissemination of clinical practice guidelines in the Brazilian Unified Health System? Report of a pilot project
Source: Health Res Policy Syst. 2023 Mar 23;21:22. doi: 10.1186/s12961-023-00966-y (PMC10035473; doi:10.1186/s12961-023-00966-y)
Supplement: Supplementary file 2 — Additional file 2: Table S2. Subdomains and attributes used in the standardization of the format for clinical guidelines. [file 12961_2023_966_MOESM2_ESM.docx]

**Additional file 2** - Subdomains and attributes used in the standardization of the format for clinical guidelines

| **Subdomain** | **Main attributes** |
| --- | --- |
| Multiple versions of the guidelines (1, 2, 5) | - Use of multiple formats or alternative versions  - Adaptation of the guidelines according to the preferences of the target audience  - Focus on end users  - Details provided at various levels of granularity  - Modalities: electronic (dynamic and static) and non -electronic  - Accessibility |
| Document components (2) | - Specific components included in the guidelines  - Summaries or algorithms with links to more extensive explanations of the guidelines  - Inclusion of important components for implementation |
| Presentation: structure and organization of the text (1, 2, 5) | - Appropriate structure (include the categorization of recommendations)  - Structure guided by practice in the real world  - Sequential arrangement/grouping. |
| Presentation: document layout (1,2-5) | 1) Document size  - short version: one to two pages maximum  - long version  2) Organization of visual elements  3) Color  Use of color to organize the text; a color code can be used to combine the text with tables or graphs |
| Presentation of information (2-5) | Textual information  - Framework  - Liveliness  - Depth of field  - Evaluability |
|  | Non-textual information  - Forms of display: tables, algorithms, photos, graphs |

Source: the authors

1. Kastner M, Makarski J, Hayden L, Durocher L, Chatterjee A, Brouwers M, Bhattacharyya O. Making sense of complex data: a mapping process for analyzing findings of a realist review on guideline implementability. BMC Med Res Methodol. 2013 Sep 12;13:112. doi: 10.1186/1471-2288-13-112. PMID: 24028286; PMCID: PMC3848005.

2. Kastner M, Bhattacharyya O, Hayden L, Makarski J, Estey E, Durocher L, Chatterjee A, Perrier L, Graham ID, Straus SE, Zwarenstein M, Brouwers M. Guideline uptake is influenced by six implementability domains for creating and communicating guidelines: a realist review. J Clin Epidemiol. 2015 May;68(5):498-509. doi: 10.1016/j.jclinepi.2014.12.013. Epub 2015 Jan 10. PMID: 25684154.

3. Grudniewicz A, Bhattacharyya O, McKibbon KA, Straus SE. User-Centered Design and Printed Educational Materials: A Focus Group Study of Primary Care Physician Preferences. J Contin Educ Health Prof. 2016 Fall;36(4):249-255. doi: 10.1097/CEH.0000000000000112. PMID: 28350305.

4. Grudniewicz A, Bhattacharyya O, McKibbon KA, Straus SE. Redesigning printed educational materials for primary care physicians: design improvements increase usability. Implement Sci. 2015 Nov 4;10:156. doi: 10.1186/s13012-015-0339-5. PMID: 26537589; PMCID: PMC4634785.

5. Gupta S, Rai N, Bhattacharrya O, Cheng AYY, Connelly KA, Boulet LP, Kaplan A, Brouwers MC, Kastner M. Optimizing the language and format of guidelines to improve guideline uptake. CMAJ. 2016 Oct 4;188(14):E362-E368. doi: 10.1503/cmaj.151102. Epub 2016 Apr 18. PMID: 27091799; PMCID: PMC5047836.
