# Supplementary material for: How to improve the dissemination of clinical practice guidelines in the Brazilian Unified Health System? Report of a pilot project
Source: Health Res Policy Syst. 2023 Mar 23;21:22. doi: 10.1186/s12961-023-00966-y (PMC10035473; doi:10.1186/s12961-023-00966-y)
Supplement: Supplementary file 3 — Additional file 3. Example of a CPG, full version. [file 12961_2023_966_MOESM3_ESM.pdf]

MINISTÉRIO DA SAÚDE

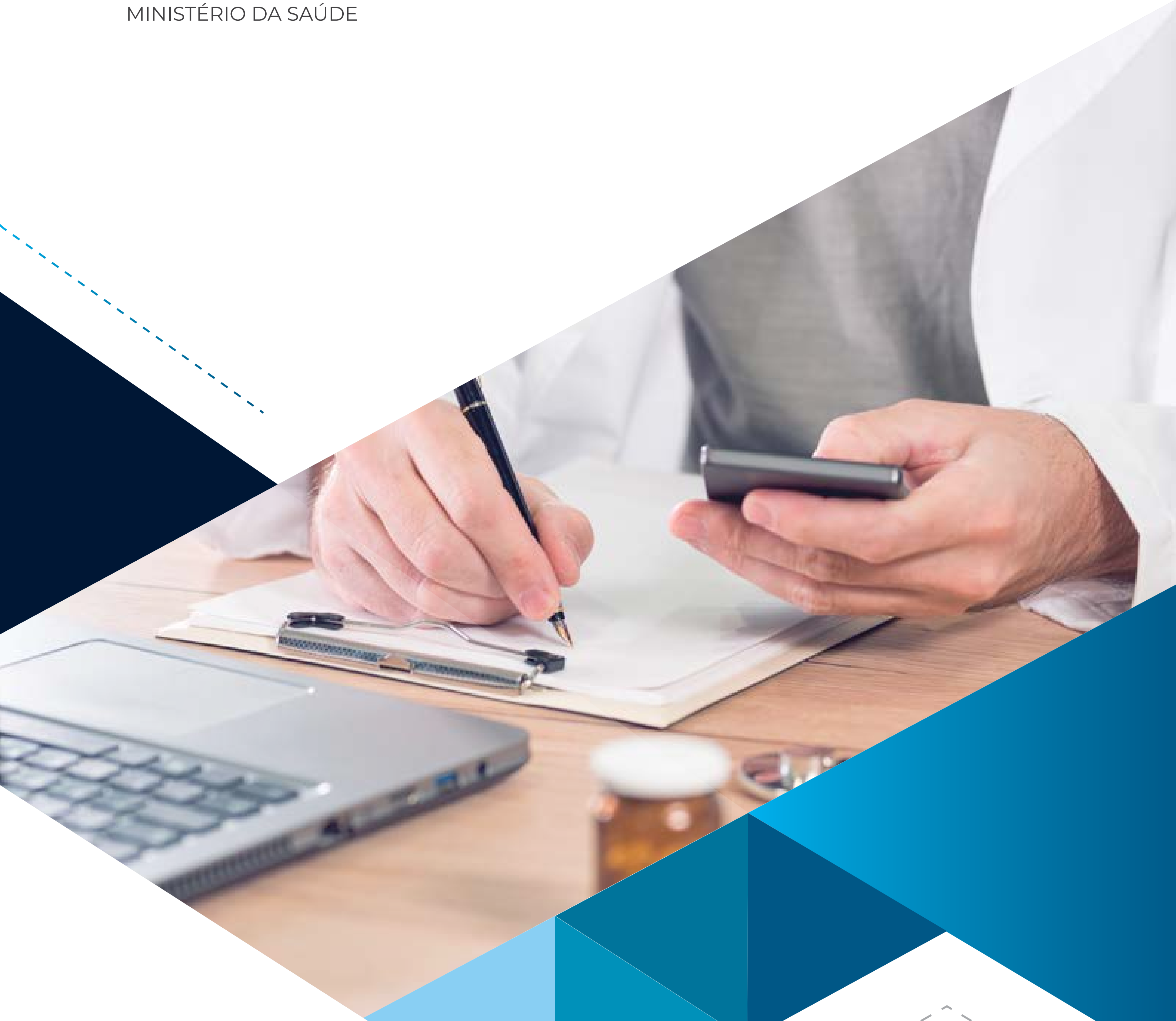

PROTOCOLO CLÍNICO E DIRETRIZES TERAPÊUTICAS DA COLANGITE BILIAR PRIMÁRIA

BRASÍLIA – DF
2020

DISTRIBUIÇÃO GRATUITA
VENDA PROIBIDA

MINISTÉRIO DA SAÚDE
Secretaria de Ciência, Tecnologia, Inovação e Insumos Estratégicos em Saúde
Departamento de Gestão e Incorporação de Tecnologias e Inovação em Saúde

PROTOCOLO CLÍNICO E DIRETRIZES TERAPÊUTICAS DA COLANGITE BILIAR PRIMÁRIA

BRASÍLIA – DF
2020

DISTRIBUIÇÃO GRATUITA
VENDA PROIBIDA

2020 Ministério da Saúde.

Esta obra é disponibilizada nos termos da Licença CreativeCommons – Atribuição – Não Comercial – Compartilhamento pela mesma licença 4.0 Internacional. É permitida a reprodução parcial ou total desta obra, desde que citada a fonte.

A coleção institucional do Ministério da Saúde pode ser acessada, na íntegra, na Biblioteca Virtual em Saúde do Ministério da Saúde: www.saude.gov.br/bvs.

Tiragem: 1ª edição – 2020 – versão eletrônica

Elaboração, distribuição e informações:
MINISTÉRIO DA SAÚDE
Secretaria de Ciência, Tecnologia, Inovação e Insumos Estratégicos em Saúde
Departamento de Gestão e Incorporação de Tecnologias e Inovação em Saúde
Coordenação de Gestão de Protocolos Clínicos e Diretrizes Terapêuticas
Esplanada dos Ministérios, bloco G, Edifício Sede, 8º andar
CEP: 70058-900 – Brasília/DF
Tel.: (61) 3315-2848
Site: http://conitec.gov.br/
E-mail: conitec@saude.gov.br

Elaboração:
Coordenação de Gestão de Protocolos Clínicos e Diretrizes Terapêuticas – CPCDT/CGGTS/DGITIS/SCTIE/MS

Comitê gestor:
Departamento de Gestão e Incorporação de Tecnologias e Inovação em Saúde – DGITIS
Coordenação de Gestão de Protocolos Clínicos e Diretrizes Terapêuticas – CPCDT
Secretaria de Atenção Especializada à Saúde – SAES

Grupo elaborador:
Camila Francisca Tavares Chacarolli – CPCDT/DGITIS
Carolina Maria Teixeira Saquis Neves – CPCDT/DGITIS
Daniel Zanetti Scherrer – CPCDT/DGITIS
Rodolfo Rêgo Deusdará Rodrigues – CPCDT/DGITIS
Verónica Elizabeth Mata – CPCDT/DGITIS

Colaboração externa:
Sociedade Brasileira de Hepatologia

Revisão:
Coordenação de Gestão de Protocolos Clínicos e Diretrizes Terapêuticas – CPCDT
Subcomissão Técnica de Protocolos Clínicos e Diretrizes Terapêuticas
Jorgiany Souza Emerick Ebeidalla – CPCDT/CGGTS/DGITIS/SCTIE/MS
Sarah Nascimento Silva – CPCDT/CGGTS/DGITIS/SCTIE/MS

Projeto gráfico:
Gustavo Veiga e Lins

Projeto de diagramação:
Fiocruz Brasília - projeto 'Apoio ao aprimoramento da gestão de tecnologias no SUS por meio da plataforma de tradução, intercâmbio e apropriação social do conhecimento', financiado pelo Departamento de Gestão e Incorporação de Tecnologias e Inovação em Saúde da Secretaria de Ciência, Tecnologia e Insumos Estratégicos do Ministério da Saúde (DGITIS/SCTIE/MS), através do Termo de Execução Descentralizada nº 43/2016.

Coordenação-geral do projeto de diagramação:
Jorge Otávio Maia Barreto

Coordenação dos subprojetos de diagramação:
Everton Nunes da Silva
Maria Sharmila Alina de Sousa
Viviane Cássia Pereira

Supervisão:
Clementina Corah Lucas Prado – CGGTS/DGITIS/SCTIE/MS
Maria Inez Pordeus Gadelha – SAES/MS
Vania Cristina Canuto Santos – DGITIS/SCTIE/MS

Normalização:
Daniela Ferreira Barros da Silva – Editora MS/CGDI

Ficha Catalográfica

Brasil. Ministério da Saúde. Secretaria de Ciência, Tecnologia, Inovação e Insumos Estratégicos em Saúde. Departamento de Gestão e Incorporação de Tecnologias e Inovação em Saúde.

Protocolo Clínico e Diretrizes Terapêuticas da Colangite Biliar Primária [recurso eletrônico] / Ministério da Saúde, Secretaria de Ciência, Tecnologia, Inovação e Insumos Estratégicos em Saúde, Departamento de Gestão e Incorporação de Tecnologias e Inovação em Saúde. – Brasília : Ministério da Saúde, 2020.

21 p.

Este Protocolo Clínico e Diretrizes Terapêuticas compreende o conteúdo da Portaria Conjunta SAES/SCTIE nº 11, de 09 de setembro de 2019.
Modo de acesso: World Wide Web: http://bvsms.saude.gov.br/bvs/publicacoes/protocolo_clinico_terapeuticas_colangite_biliar.pdf
ISBN 978-85-334-2801-0

1. Protocolos clínicos. 2. Colangite. 3. Cirrose hepática biliar. I. Título.

CDU 616.36-004

Catalogação na fonte – Coordenação-Geral de Documentação e Informação – Editora MS – OS 2020/0171

Título para indexação:
Clinical Practice Guidelines Primary Biliary Cholangitis

SUMÁRIO

1 INTRODUÇÃO ---------- 4
2 CLASSIFICAÇÃO ESTATÍSTICA INTERNACIONAL DE DOENÇAS E PROBLEMAS RELACIONADOS À SAÚDE (CID-10) ---------- 5
3 DIAGNÓSTICO ---------- 5
3.1 Diagnóstico clínico ---------- 5
3.2 Imagem ---------- 5
3.3 Diagnóstico laboratorial ---------- 6
3.3.1 Marcadores bioquímicos ---------- 6
3.3.2 Marcadores imunológicos ---------- 6
3.3.3 Métodos histológicos ---------- 6
4 CRITÉRIOS DE INCLUSÃO ---------- 9
5 CRITÉRIOS DE EXCLUSÃO ---------- 9
6 CASOS ESPECIAIS ---------- 10
7 TRATAMENTO ---------- 10
7.1 Tratamento não medicamentoso ---------- 10
7.2 Tratamento medicamentoso - ácido ursodesoxicólico ---------- 10
8 FÁRMACOS ---------- 11
8.1 Esquema de administração ---------- 11
8.2 Contraindicações ---------- 12
9 TEMPO DE TRATAMENTO – CRITÉRIOS DE INTERRUPÇÃO ---------- 12
10 BENEFÍCIOS ESPERADOS ---------- 12
11 MONITORIZAÇÃO ---------- 12
12 REGULAÇÃO/CONTROLE/AVALIAÇÃO PELO GESTOR ---------- 13
13 TERMO DE ESCLARECIMENTO E RESPONSABILIDADE (TER) ---------- 14
REFERÊNCIAS ---------- 14
APÊNDICE – METODOLOGIA DE BUSCA E AVALIAÇÃO DA LITERATURA ---------- 17
ANEXO – TERMO DE ESCLARECIMENTO E RESPONSABILIDADE ---------- 19

1 INTRODUÇÃO

A colangite biliar primária (CBP), anteriormente denominada cirrose biliar primária, é uma doença hepática colestática crônica progressiva, autoimune com reatividade sorológica para anticorpos antimitocondriais (AAM) e anticorpo antinúcleo (AAN), caracterizada pela inflamação e destruição progressiva dos ductos biliares interlobulares de pequeno e médio calibre. Apresenta evidência histológica de colangite crônica não-supurativa, granulomatosa, linfocitária de ductos biliares de pequeno calibre (1-3).

A etiologia da doença não é clara, contudo, interações imunogenéticas e ambientais (2) parecem desencadear uma intensa resposta autoimune contra as células epiteliais biliares, levando à lesões hepato-biliares (4).

A CBP deve ser suspeitada em pacientes com anormalidades colestáticas persistentes, por mais de 6 meses, em testes bioquímicos séricos hepáticos, ou sintomas como fadiga e prurido (1). Trata-se de um distúrbio hepático raro que afeta predominantemente as mulheres (10:1), com a mediana de idade do início da doença de 50 anos (5) e raros relatos de caso na adolescência (6,7). Os pacientes mais jovens são os mais afetados, com comprometimento da qualidade de vida devido aos sintomas da doença. Neste sentido, a menor capacidade funcional imposta pela fadiga e as implicações sociais do prurido, limitam a vida social destes pacientes (8). Esta doença pode evoluir para cirrose, doença hepática terminal ou a morte (1,2,9).

No momento do diagnóstico cerca de metade dos pacientes são assintomáticos (10,11). Entretanto, mesmo em fase assintomática, o tratamento pode retardar a progressão e melhorar a sobrevida do paciente livre de transplante (12).

Uma revisão sistemática que avaliou estudos epidemiológicos de base populacional de diversos países, apontou incidência variando de 0,33 a 5,8 por 100 mil habitantes e prevalência variando de 1,91 a 40,2 por 100 mil habitantes, evidenciando grande incerteza nestes parâmetros (13). Todavia, atualmente não existem estudos que descrevam dados epidemiológicos para a CBP na população brasileira.

Neste contexto, o presente documento tem como objetivo orientar os profissionais de saúde quanto ao diagnóstico e tratamento adequado da CBP a fim de evitar complicações.

A identificação da doença em seu estágio inicial e o encaminhamento ágil e adequado para o atendimento especializado dão à Atenção Primária um caráter essencial para um melhor resultado terapêutico e prognóstico dos casos.

Este Protocolo visa a estabelecer os critérios diagnósticos e terapêuticos da colangite biliar primária. A metodologia de busca e avaliação das evidências estão detalhadas no **Apêndice 1**.

2 CLASSIFICAÇÃO ESTATÍSTICA INTERNACIONAL DE DOENÇAS E PROBLEMAS RELACIONADOS À SAÚDE (CID-10)

K74.3	Cirrose biliar primária (Colangite destrutiva não-supurativa crônica)

3 DIAGNÓSTICO

O diagnóstico de CBP é baseado em achados clínicos, laboratoriais e exames de imagem.

3.1 Diagnóstico clínico

O paciente típico com doença sintomática é mulher de meia idade com queixas de fadiga e prurido. Outros sinais e sintomas, incluem dor abdominal no quadrante superior direito, dor, anorexia, hiperpigmentação, xantelasma, hepatoesplenomegalia, icterícia, alteração no padrão do sono, osteoporose e síndrome de má-absorção. A icterícia costuma ocorrer no curso mais avançado da doença (14).

Outras doenças autoimunes podem ser vistas com uma maior frequência em pacientes com CBP, como síndrome de Sjögren (SICCA síndrome), esclerodermia, síndrome de CREST, tireoidite, artrite, síndrome de Raynaud, doença celíaca e lúpus eritematoso sistêmico (15,16).

O diagnóstico da CBP deve ser suspeitado nos pacientes com colestase crônica, por mais de 6 meses, depois da exclusão de outras causas de doença do fígado, particularmente em mulheres com elevação inexplicada de fosfatase alcalina sérica(FA) (17).

Os diagnósticos diferenciais incluem colestase medicamentosa, obstrução biliar, sarcoidose, hepatite autoimune, colangite esclerosante primária (17) e hepatites virais (18).

3.2 Imagem

A avaliação não-invasiva por imagem do fígado e da árvore biliar é mandatória em todos os pacientes com evidência bioquímica de colestase (17). A ausência de dilatação do ducto biliar indica que a causa da colestase deve ser intra-hepática, enquanto que a dilatação dos ductos sugere a avaliação de causas extra-hepáticas (14). Usualmente, a ultrassonografia é o primeiro exame a ser feito para excluir obstrução extra-hepática biliar, que é parte do diagnóstico diferencial da CBP (15). Pode-se também usar tomografia computadorizada ou ressonância magnética (14).

A colangioressonância por ressonância magnética é um exame seguro e com acurácia para detecção de estenose e dilatação de ductos intra- e extra-hepáticos, sendo fundamental para diagnóstico de colangite esclerosante primária ou secundária (1). A colangiopancreatografia retrógrada por via endoscópica é clinicamente equivalente na detecção de lesões e litíase por ductos biliares causada por obstrução extra-hepática, particularmente no ducto biliar distal (1).

3.3 Diagnóstico laboratorial

O diagnóstico de CBP envolve métodos bioquímicos, imunológico e, eventualmente, histológicos. A confirmação diagnóstica é realizada na presença de 2 dos 3 critérios apresentados a seguir (1,19,20) e de acordo com o fluxograma de Diagnóstico de Colangite Biliar Primária abaixo.

- Elevação de enzimas colestáticas, especialmente da FA;
- Presença de anticorpos antimitocondriais (AAM) ou anticorpo antinúcleo (AAN) por imunoflourescencia indireta, padrões nucleares tipo membrana nuclear e nuclear pontilhado com pontos isolados ou por Elisa, anti- gp210 e anti-sp-100 (1,17);
- Evidência histológica de colangite destrutiva não-supurativa.

3.3.1 Marcadores bioquímicos

No início do curso da doença, a CBP é caracterizada por níveis séricos elevados de FA e gama-glutamiltransferase (GGT), enquanto no curso mais tardio da doença há níveis séricos elevados de bilirrubina e diminuição da albumina sérica, sendo considerados como importantes marcadores (18). A elevação da FA e da bilirrubina correlaciona-se com a progressão da doença, enquanto valores mais baixos são preditores de maior sobrevida e de menor necessidade de transplante hepático (21).

Quase todos os pacientes têm aumento de FA, tipicamente ≥ 1,5 vezes o limite superior da normalidade, e elevação de GGT (14,15). As aminotransferases/transaminases séricas, aspartato- aminotransferase (AST/TGO) e alanino-aminotransferase (ALT/TGP), estão levemente aumentadas (geralmente, <3 vezes o limite superior normal). Elevações de AST e ALT acima de 5 vezes o limite superior da normalidade são incomuns e sugerem síndrome de sobreposição de hepatite autoimune e CBP ou coexistência de hepatites virais (14).

Uma queda na albumina sérica e na contagem de plaquetas são indicadores do desenvolvimento de cirrose e de hipertensão portal (17).

3.3.2 Marcadores imunológicos

Em pacientes com colestase intra-hepática crônica, a pesquisa de AAM é útil para confirmação diagnóstica. Títulos séricos elevados são detectados em cerca de 95% dos doentes, a reatividade AAM é classicamente estudada por imunofluorescência indireta ou por ELISA. Não há evidências de superioridade de uma técnica sobre a outra e a técnica escolhida é baseada na experiência local e na disponibilidade (18). A titulação do AAM é considerada positiva quando for ≥1/40 (16, 17, 22, 23). Indivíduos assintomáticos, AAM positivos, podem eventualmente desenvolver a doença (2).

Em indivíduos com AAM negativo, recomenda-se pesquisa de AAN, que pode ser realizada por imunofluorescencia indireta, padrões nucleares tipo membrana nuclear (*perinuclear rims*) e nuclear pontilhado com pontos isolados (*nuclear dots)* ou por Elisa, gp210 e sp-100, os quais são encontrados em até 30%-50% dos pacientes com CBP (16,23).

3.3.3 Métodos histológicos

A biópsia hepática é recomendada para o diagnóstico de CBP em indivíduos com AAM negativo e AAN (padrões específicos de CBP) negativo, colangiorressonância ou colangiopancreatografia retrógrada por via endoscópica normais, com quadro clínico sugestivo de CBP, conforme o fluxograma de diagnóstico de CBP. Em pacientes com AAM positivos e bioquímica hepática colestática, a biópsia não é recomen-

dada, visto que não aumenta a acurácia diagnóstica (18, 23). Na prática clínica, a maior parte dos pacientes é diagnosticada clínica e laboratorialmente de forma adequada e segura sem a necessidade da biópsia hepática (18). Ainda, a biópsia, pode ser útil para a avaliação da atividade e estadiamento da doença. As lesões histológicas da CBP classicamente dividem-se em quatro estágios, como proposto por Ludwig *et al.* (24) e Scheuer (25), de acordo com o grau de lesão do canal biliar, inflamação e fibrose. O Quadro 1 demonstra o sistema de estadiamento das lesões histológicas da CBP (22).

Quadro 1 – Estadiamento das lesões histológicas da CBP

Estadiamento	Scheuer	Ludwig
I (Portal)	Inflamação portal com lesão do ducto biliar, contendo ou não lesão ductal florida	Inflamação portal com lesão do ducto biliar, contendo ou não lesão ductal florida
II (Periportal)	Reação ductular (fibrose periportal presente)	Inflamação periportal (fibrose periportal presente)
III (Septal)	Fibrose em ponte (ductopenia normalmente presente)	Fibrose em ponte (ductopenia normalmente presente)

Fonte: Autoria própria.

Fluxograma de Diagnóstico de Colangite Biliar Primária (CBP)

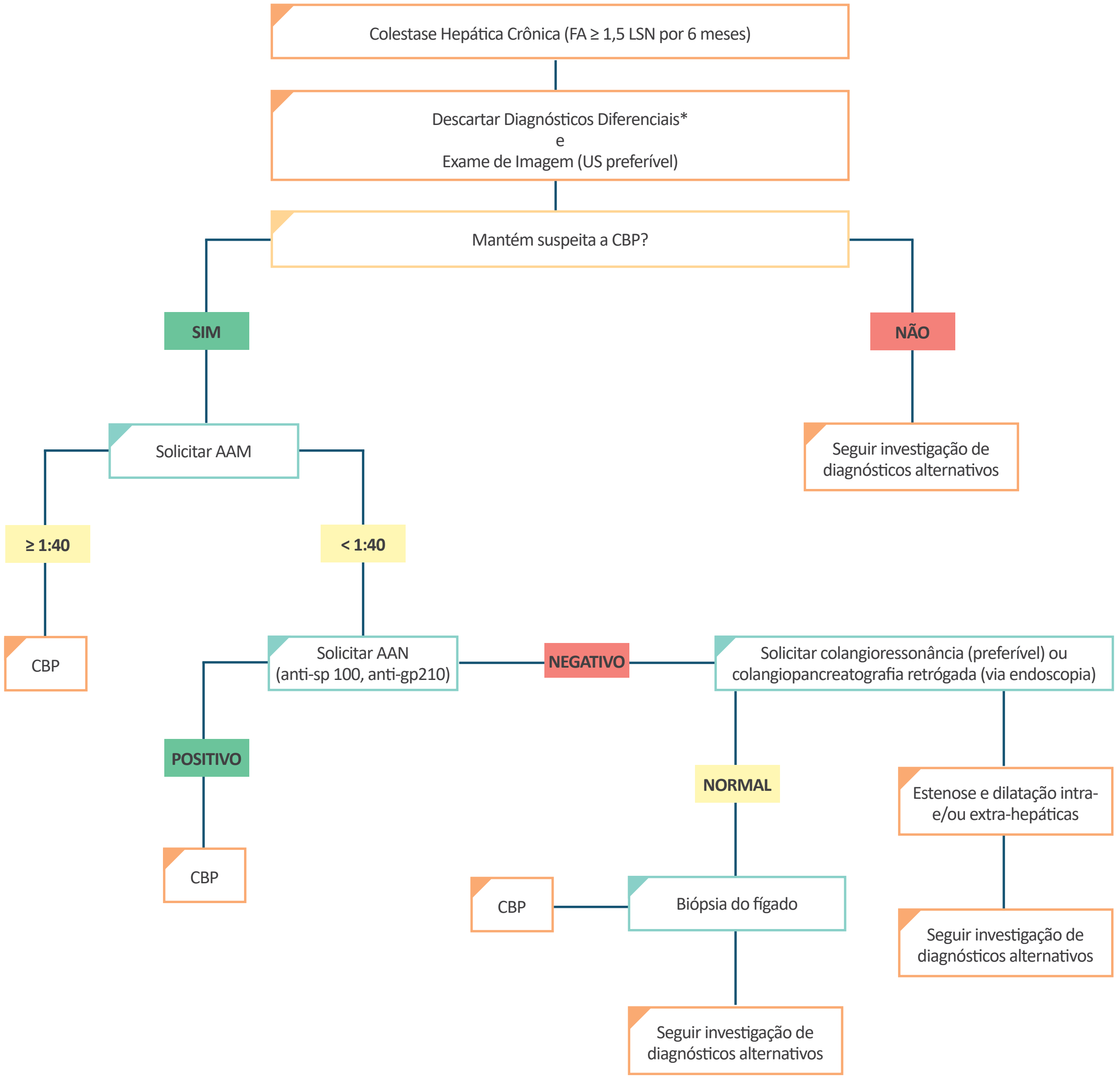

* Diagnósticos especiais:
Colestase medicamentosa,
Hepatites virais,
Obstrução biliar,
Sarcoidose,
Hepatite autoimune,
Colangite esclerosante primária.

Legenda: FA, fosfatase alcalina; LSN, limite superior da normalidade; AAM, anticorpo antimitocôndria; AAN, anticorpo antinúcleo; CBP, colangite biliar primária.

4 CRITÉRIOS DE INCLUSÃO

Serão incluídos neste Protocolo pacientes com diagnóstico de CBP, de acordo com o item 3 de diagnóstico, de ambos os sexos, que preencham pelo menos, 2 de 3 dos seguintes critérios diagnósticos:

Elevação de enzimas colestáticas, especialmente da FA

Presença de AAM ou AAN (padrões específicos à imunofluorescência ou anti-gp210 e anti-sp-100 por Elisa)

Evidência histológica de colangite destrutiva não-supurativa

5 CRITÉRIOS DE EXCLUSÃO

Serão excluídos do tratamento com o ácido ursodesoxicólico os pacientes que se enquadrem nas seguintes situações:

Alergia a ácido ursodesoxicólico ou a qualquer um dos componentes da formulação

Úlcera péptica (gástrica ou duodenal) em fase ativa

Doença intestinal inflamatória e outras condições do intestino delgado, cólon e fígado, que possam interferir com a circulação entero-hepática dos sais biliares (ressecção ileal e estoma, colestase intra e extra-hepática, doença hepática grave)

Cólicas biliares frequentes

Inflamação aguda da vesícula biliar ou trato biliar

Oclusão do trato biliar (oclusão do ducto biliar comum ou um ducto cístico)

Contratilidade comprometida da vesícula biliar

Cálculos biliares calcificados radiopacos

Pacientes com intolerância a galactose, deficiência de Lapp lactase ou má absorção de glicose galactose

6 CASOS ESPECIAIS

A evidência científica apontada na literatura para tratamento de gestantes com CBP com ácido ursodesoxicólico é limitada a série de casos (26). Portanto, recomenda-se a utilização de métodos contraceptivos em mulheres em idade fértil com diagnóstico de CBP. Em gestantes, quando o tratamento medicamentoso for claramente necessário, o hepatologista deve ser consultado e o tratamento deve ser individualizado.

7 TRATAMENTO

7.1 Tratamento não medicamentoso

Os pacientes com CBP devem ser orientados a suspender o consumo de álcool ou tabaco e evitar o uso alimentos e bebidas com a presença de cafeína. Deve ser incentivada prática de exercícios físicos regulares, e a adoção de uma dieta balanceada (10).

Pacientes com prurido intratável grave, resistência ao tratamento medicamentoso e complicações cirróticas devem ser avaliados para receber transplante hepático. O transplante de hepático é eficaz, mas só deve ser considerado quando as demais intervenções disponíveis mostrarem ineficácia.

O transplante hepático melhorou drasticamente a sobrevida em doentes com CBP em estádio avançado. As indicações para transplante hepático em pacientes com CBP são as mesmas que para pacientes com outras etiologias de insuficiência hepática (27-29). As taxas de sobrevida são superiores a 90% e, após cinco anos, as taxas de sobrevida são de 80%-85% (30).

Estes doentes devem ser encaminhados para um centro de transplante hepático, habilitado pelo SUS, conforme preconizado na Portaria nº 2.600, de 21 de outubro de 2009.

7.2 Tratamento medicamentoso – ácido ursodesoxicólico

O primeiro tratamento aprovado pela agência *U.S. Food and Drug Administration* para CBP foi o ácido Ursodesoxicólico (15). O ácido ursodesoxicólico é um ácido biliar hidrofílico que inibe a síntese hepática do colesterol e estimula a síntese de ácidos biliares, reestabelecendo o equilibro entre eles. Deste modo, o medicamento possui propriedades citoprotetoras, coleréticas, imunomoduladoras, anti-in-

flamátorias, baixa propriedade hepatotóxica, ou seja, é seguro (10). O tratamento com ácido ursodesoxicólico é considerado o esquema de primeira linha para todos os pacientes com CBP e usualmente é mantido por vida toda. O medicamento pode ser utilizada em qualquer estágio da CBP, desde que as enzimas hepáticas colestáticas sejam anormais (1). Pacientes que são diagnosticados em idade mais precoce (<50 anos) tem maior probabilidade de apresentar sintomas mais intensos, diminuição da qualidade de vida, menor probabilidade de responder ao tratamento com ácido ursodesoxicólico e apresentam um curso mais agressivo da doença (31).

As evidências que fundamentam a eficácia e segurança deste medicamento são encontradas em revisões sistemáticas de ensaios clínicos randomizados e estudos de coorte.

Os estudos não evidenciaram diferença estatisticamente significantes para os desfechos eventos adversos graves e mortalidade quando comparado ao placebo/não tratamento. O ácido ursodesoxicólico é bem tolerado e seguro com mínimos efeitos adversos. O efeito adverso mais comum é ganho de peso. Outros efeitos adversos mais comuns da ácido ursodesoxicólico: diarreia e fezes pastosas. Outros efeitos adversos mais raros (ocorre em menos de 0,01% dos pacientes que utilizam este medicamento): intensa dor abdominal superior direita durante tratamento de colangite biliar primária; descompensação hepática foi observada em terapia de estágios avançados de cirrose biliar primária que regrediu parcialmente após a descontinuidade do tratamento; urticária; calcificação de cálculos (32). Já com relação à eficácia, os estudos apontaram sobrevida global maior no grupo tratado com ácido ursodesoxicólico (33).

A sobrevida livre de transplante no acompanhamento em longo prazo foi maior no grupo de pacientes tratados com ácido ursodesoxicólico a partir do quinto ano de tratamento, com resultados estatisticamente significantes para os anos 5, 8 e 10 ($p < 0,01$) (33).

Aproximadamente 65% dos pacientes com CBP respondem integralmente ao tratamento com ácido ursodesoxicólico e têm uma expectativa de vida normal (34).

8 FÁRMACOS

Ácido ursodesoxicólico: comprimido de 50 mg, 150 mg e 300 mg.

8.1 Esquema de administração

Ácido ursodesoxicólico: 13 mg a 15 mg/kg/dia, via oral, dose única ou dividida em até quatro doses diárias

Caso seja necessário o uso concomitante de substâncias que inibem a absorção intestinal de ácidos biliares, como a colestiramina, colestipol ou antiácidos à base de alumínio, estas devem ser tomadas ao menos 2 horas antes ou após o ácido ursodesoxicólico (32).

8.2 Contraindicações

Efeitos colaterais são mínimos e infrequentes, havendo relatos de pequeno ganho de peso no primeiro ano de tratamento e efeitos gastrointestinais (10).

9 TEMPO DE TRATAMENTO – CRITÉRIOS DE INTERRUPÇÃO

O tempo de tratamento não pode ser pré-determinado e deve ser realizado a longo prazo. Espera-se benefícios em termos de retardo ou interrupção da evolução da doença, quando realizado de maneira continua (35).

10 BENEFÍCIOS ESPERADOS

Espera-se com este Protocolo o diagnóstico ágil para os pacientes com CBP e o tratamento adequado contribua para a redução dos sintomas, prurido e fadiga, por meio de diagnóstico precoce e reversão ou regressão das lesões hepáticas, aumento na sobrevida livre de transplante, melhora dos sintomas e da qualidade de vida dos pacientes.

11 MONITORIZAÇÃO

A resposta bioquímica ao tratamento é avaliada após um ano, depois do início do tratamento com testes bioquímicos hepáticos, mais especificamente FA e bilirrubina total, usando um dos critérios estabelecidos no Quadro 2 (17). O paciente que não respondeu adequadamente deve ser avaliado em relação à adesão terapêutica e quanto à possibilidade de outros diagnósticos associados, tais como síndrome de sobreposição ou doença celíaca (16,18).

As medidas FA e bilirrubinas são duas fortes variáveis usadas para predizer prognóstico.

Mais da metade dos pacientes, 60%, tratados apresentam melhora nos marcadores bioquímicos, a qual pode ser observada entre um e seis meses após o início, sendo que a maioria dos casos responde ao tratamento ainda nos primeiros três meses (10).

Quadro 2 – Avaliação da resposta ao tratamento com ácido ursodesoxicólico em pacientes com CBP1

Definições binárias qualitativas	Tempo (meses)	Falha ao tratamento
Barcelona	12	Redução < 40% na ALP e ALP ≥ 1 x LSN
Paris I	12	ALP ≥ 3 x LSN ou AST ≥2 x LSN ou bilirrubina >1mg/dl
Roterdã	12	Bilirrubina ≥1 x LSN ou albumina <1 x LSN
Toronto	24	ALP >1,67 x LSN
Paris II	12	ALP≥1,5 x LSN ou AST≥1,5 x LSN ou bilirrubina > 1mg/dl
**Sistema de escores contínuos**	**Tempo (meses)**	**Parâmetros de pontuação**
UK-CBP	12	Bilirrubina, ALP e AST (ou ALT) em 12 meses. Albumina e contagem de plaquetas no baseline
GLOBE	12	Bilirrubina, ALP, albumina, contagem de plaquetas em 12 meses. Idade no baseline.

Fonte: Autoria própria.
Legenda: GGT: gama-glutamiltransferase; ALP: fosfatase alcalina; AST/TGO: aspartato aminotranferase; LSN: limite superior do normal.

Alguns pacientes podem apresentar piora do quadro clínico no início do tratamento, por exemplo, piora do prurido ou diarreia. Neste caso, a dose do ácido ursodesoxicólico deve ser reduzida até a melhora dos sintomas e gradualmente aumentado de acordo com a tolerância do paciente. Em caso de persistência dos sintomas a terapia deve ser descontinuada (32).

12 REGULAÇÃO/CONTROLE/AVALIAÇÃO PELO GESTOR

Devem ser observados os critérios de inclusão e exclusão de pacientes neste Protocolo, a duração e a monitorização do tratamento, bem como a verificação periódica das doses prescritas e dispensadas e a adequação de uso do medicamento.

Pacientes com CBP devem ser atendidos em serviços especializados, preferentemente em centros de referência em hepatologia, para seu adequado diagnóstico, tratamento e acompanhamento.

Verificar na Relação Nacional de Medicamentos Essenciais (RENAME) vigente em qual componente da Assistência Farmacêutica se encontra o medicamento preconizado neste Protocolo.

13 TERMO DE ESCLARECIMENTO E RESPONSABILIDADE (TER)

Deve-se informar ao paciente, ou seu responsável legal, sobre os potenciais riscos, benefícios e efeitos adversos relacionados ao uso do medicamento preconizado neste PCDT, bem como critérios para interrupção do tratamento, levando-se em consideração as informações contidas no TER.

REFERÊNCIAS

1. EUROPEAN ASSOCIATION FOR THE STUDY OF THE LIVER. EASL. Clinical Practice Guidelines: The diagnosis and management of patients with primary biliary cholangitis. **Journal of Hepatology**, Copenhagen, v. 67, n. 1, p. 145-172, July 2017.
2. LIBERAL, R.; GRANT, C. R. Cirrhosis and autoimmune liver disease: current understanding. **World Journal of Hepatology**, *[s. l.]*, v. 8, n. 28, p. 1157-1168, Oct. 2016.
3. HARINCK, F. Primary biliary cholangitis, let's try to keep the new nomenclature correct! **The Netherlands Journal of Medicine**, *[s. l.]*, v. 74, n. 6, p. 229-230, July 2016.
4. RUDIC, J. S. *et al.* Ursodeoxycholic acid for primary biliary cirrhosis. **The Cochrane Database of Systematic Reviews**, Oxford, 2012, n. 12, p. CD000551, Dec. 2012. Disponível em: https://www.ncbi.nlm.nih.gov/pmc/articles/PMC7045744/. Acesso em: 20 jul. 2020.
5. GRIFFITHS, L.; DYSON, J. K.; JONES, D. E. J. The new epidemiology of primary biliary cirrhosis. **Seminars in Liver Disease**, New York, v. 34, n. 3, p. 318-328, Aug. 2014.
6. DAHLAN, Y. *et al.* Pediatric-onset primary biliary cirrhosis. **Gastroenterology**, Philadelphia, v. 125, n. 5, p. 1476-1479, Nov. 2003.
7. KITIC, I. *et al.* Twelve-Year-Old Girl with Primary Biliary Cirrhosis. **Case Reports in Pediatrics**, New York, v. 2012, Nov. 2012. DOI 10.1155/2012/937150. Disponível em: https://www.ncbi.nlm.nih.gov/pmc/articles/PMC3515888/. Acesso em: 20 fev. 2019.
8. DYSON, J. K. *et al.* The inter-relationship of symptom severity and quality of life in 2055 patients with primary biliary cholangitis. **Alimentary Pharmacology & Therapeutics**, *[s. l.]*, v. 44, n. 10, p. 1039-1050, Nov. 2016.
9. LLEO, A. *et al.* Evolving Trends in Female to Male Incidence and Male Mortality of Primary Biliary Cholangitis. **Scientific Reports**, *[s. l.]*, v. 6, p. 25906, May 2016.
10. SOCIEDADE BRASILEIRA DE HEPATOLOGIA. **Recomendações da sociedade brasileira de hepatologia para diagnóstico e tratamento das doenças colestáticas e hepatite autoimune:** parte II. São Paulo: SBH, 2015.

11. SAFFIOTI, F. *et al.* Pharmacological interventions for primary biliary cholangitis: an attempted network meta-analysis. **The Cochrane Database of Systematic Reviews,** *[s. l.]*, v. 3, n. 3, p. CD011648, Mar. 2017.

12. GATSELIS, N. K.; DALEKOS, G. N. Molecular diagnostic testing for primary biliary cholangitis. **Expert Review of Molecular Diagnostics**, *[s. l.]*, v. 16, n. 9, p. 1001-1010, Sept. 2016.

13. BOONSTRA, K.; BEUERS, U.; PONSIOEN, C. Y. Epidemiology of primary sclerosing cholangitis and primary biliary cirrhosis: a systematic review. **Journal of Hepatology**, Copenhagen, v. 56, n. 5, p. 1181-1188, May 2012.

14. QAYED, E.; SRINIVASAN, S.; SHAHNAVAZ, N. **Sleisenger and Fordtran's Gastrointestinal and Liver Disease Review and Assessment.** 10th ed. *[S. l.]*: Elsevier, 2016.

15. BOYER, T. D.; WRIGHT, T. L.; MANNS, M. P. **Zakim and Boyer's Hepatology**. *[S. l.]*: Elsevier, 2006. DOI 10.1016/B978-1-4160-3258-8.X5001-3.

16. BITTENCOURT, P. L. *et al*. Brazilian society of hepatology recommendations for the diagnosis and management of autoimmune diseases of the liver. **Arquivos de Gastroenterologia**, São Paulo, v. 52, p. 15-46, Dec. 2015. Suplemento 1.

17. LINDOR, K. D. *et al.* Primary Biliary Cholangitis: 2018 Practice Guidance from the American Association for the Study of Liver Diseases. **Hepatology**, Baltimore, Md., v. 69, n. 1, p. 394-419, 2019.

18. HIRSCHFIELD, G. M. *et al*. The British Society of Gastroenterology/UKPBC primary biliary cholangitis treatment and management guidelines. **Gut**, *[s. l.]*, v. 67, n. 9, p. 1568-1594, Sept. 2018.

19. HUANG, Y-Q. Recent advances in the diagnosis and treatment of primary biliary cholangitis. **World Journal of Hepatology**, *[s. l.]*, v. 8, n. 33, p. 1419-1441, Nov. 2016.

20. LINDOR, K. D. *et al.* Primary biliary cirrhosis. **Hepatology**, Baltimore, Md., v. 50, n. 1, p. 291-308, July 2009.

21. NEVENS, F. *et al.* A Placebo-Controlled Trial of Obeticholic Acid in Primary Biliary Cholangitis. **The New England Journal of Medicine**, Boston, v. 375, p. 631-643, Aug. 2016.

22. ASSOCIAÇÃO EUROPÉIA PARA O ESTUDO DO FÍGADO. Recomendações de Orientação Clínica da EASL: abordagem de doenças hepáticas colestáticas. **Journal of Hepatology**, Copenhagen, v. 51, p. 237-267, 2009.

23. ZEIN, C. O.; ANGULO, P.; LINDOR, K. D. When is liver biopsy needed in the diagnosis of primary biliary cirrhosis? **Clinical Gastroenterology and Hepatology**, *[s. l.]*, v. 1, n. 2, p. 89-95, Mar. 2003.

24. LUDWIG, J.; DICKSON, E. R.; MCDONALD, G. S. Staging of chronic nonsuppurative destructive cholangitis (syndrome of primary biliary cirrhosis). **Virchows Arch**: A: Pathological Anatomy and Histology, Berlin, v. 379, n. 2, p. 103-112, Aug. 1978.

25. SCHEUER, P. J. Primary biliary cirrhosis: diagnosis, pathology and pathogenesis. **Postgraduate Medical Journal**, Oxford, v. 59, p. 106-115, 1983. Suppl. 4.

26. TRIVEDI, P. J. *et al.* Good maternal and fetal outcomes for pregnant women with primary biliary cirrhosis. **Clinical Gastroenterology and Hepatology**, *[s. l.]*, v. 12, n. 7, p. 1179-1185.e1, July 2014.

27. KUIPER, E. M. M. *et al.* Trends in liver transplantation for primary biliary cirrhosis in the Netherlands 1988-2008. **BMC Gastroenterology**, *[s. l.]*, v. 10, p. 144, Dec. 2010.

28. LEVY, C. Primary Biliary Cholangitis Guidance Update: Implications for Liver Transplantation. **Liver Transplantation**, Philadelphia, v. 24, n. 11, p. 1508-1511, Nov. 2018.

29. MACQUILLAN, G. C.; NEUBERGER, J. Liver transplantation for primary biliary cirrhosis. **Clinics in Liver Disease**, *[s. l.]*, v. 7, n. 4, p. 941-956, Nov. 2003.

30. MILKIEWICZ, P. Liver transplantation in primary biliary cirrhosis. **Clinics in Liver Disease**, *[s. l.]*, v. 12, n. 2, p. 461-472, May 2008.

31. CARBONE, M. *et al.* Sex and Age Are Determinants of the Clinical Phenotype of Primary Biliary Cirrhosis and Response to Ursodeoxycholic Acid. **Gastroenterology**, Philadelphia, v. 44, n. 3, p. 560-569, 2013. DOI 10.1053/j.gastro.2012.12.005.

32. URSACOL®. São Paulo: Zambon Laboratórios Farmacêuticos Ltda., 2015. 1 bula de remédio. Disponível em: http://www.anvisa.gov.br/datavisa/fila_bula/frmVisualizarBula.asp. Acesso em: 20 fev. 2019.

33. BRASIL. Ministério da Saúde. **Ácido ursodesoxicólico para colangite biliar primária**. Brasília, DF: MS, 2018. (Relatório de Recomendação, n. 392).

34. CORPECHOT, C. *et al.* Biochemical response to ursodeoxycholic acid and ong-term prognosis in primary biliary cirrhosis. **Hepatolology**, Baltimore, Md., v. 48, n. 3, p. 871-877, Sept. 2008.

35. DAVID, E. J. J. *et al.* **Colangite biliar primária**. 2019. Disponível em: https://bestpractice.bmj.com/topics/pt-br/344/references. Acesso em: 20 fev. 2019.

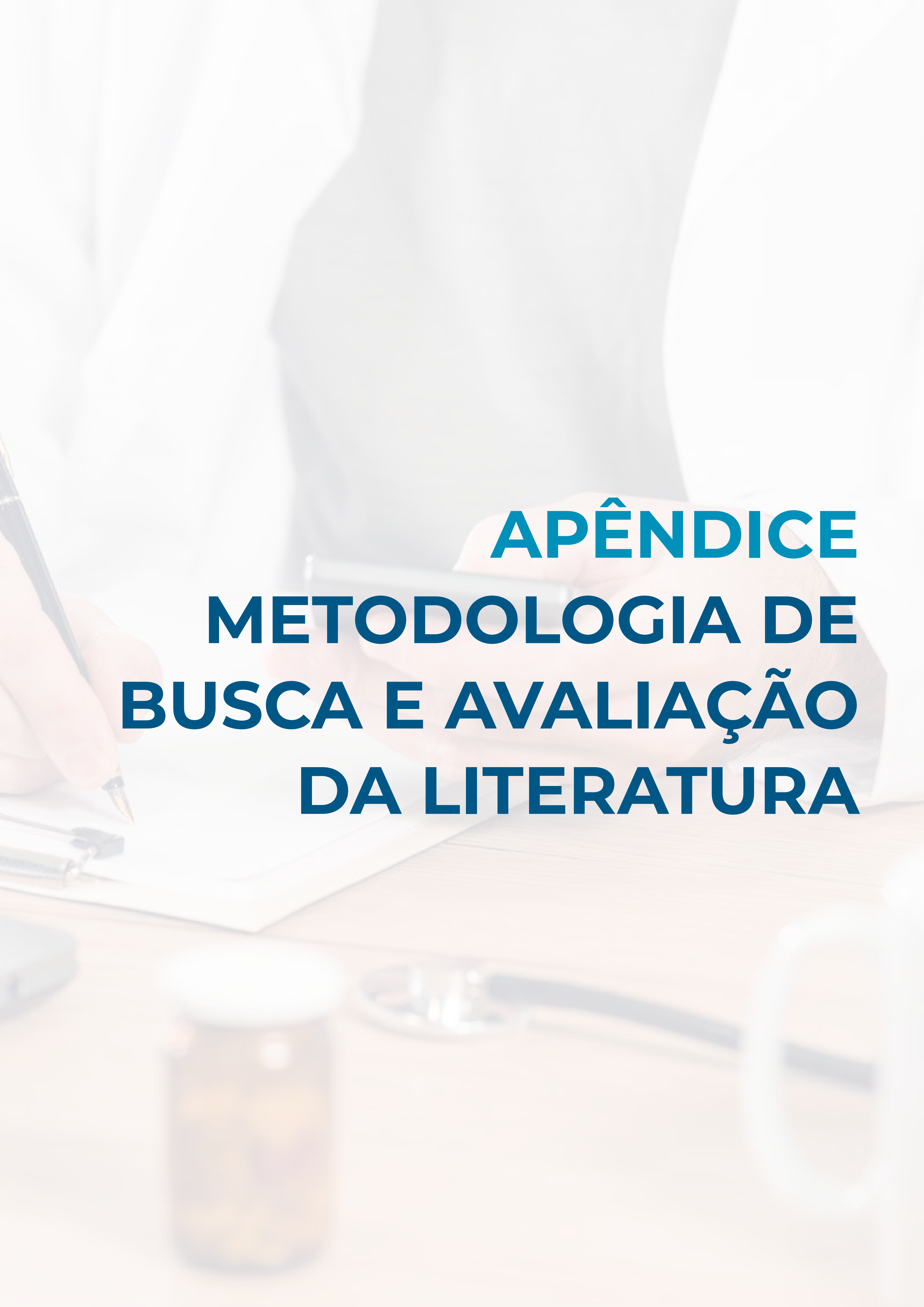

APÊNDICE
METODOLOGIA DE BUSCA E AVALIAÇÃO DA LITERATURA

METODOLOGIA DE BUSCA E AVALIAÇÃO DA LITERATURA

O processo de desenvolvimento deste Protocolo utilizou como base a Diretriz Metodológica de Elaboração de Diretrizes Clínicas do Ministério da Saúde. A Relação Nacional de Medicamentos Essenciais (RENAME) e o sítio eletrônico da Comissão Nacional de Incorporação de Tecnologias no SUS (CONITEC) foram consultados para a identificação das tecnologias disponíveis no Brasil e tecnologias demandadas ou recentemente incorporadas no SUS para o tratamento da colangite biliar primária.

A Portaria nº 47/SCTIE/MS, de 16 de outubro de 2018, tornou pública a decisão de incorporar o ácido ursodesoxicólico para o tratamento da colangite biliar primária no âmbito do SUS e, deste modo, o medicamento encontra-se incluso neste PCDT. Neste sentido, este PCDT utilizou as buscas de evidência, resultados e referências do Relatório de Recomendação nº 392/2018, ácido ursodesoxicólico para colangite biliar primária, disponível em: http://conitec.gov.br/images/Relatorios/2018/Recomendacao/Relatorio_Acidoursodesoxicolico_ ColagiteBiliar.pdf para elaborar as recomendações do tratamento medicamentoso.

Para elaboração dos demais itens deste documento foram utilizados *guidelines* internacionais e nacionais.

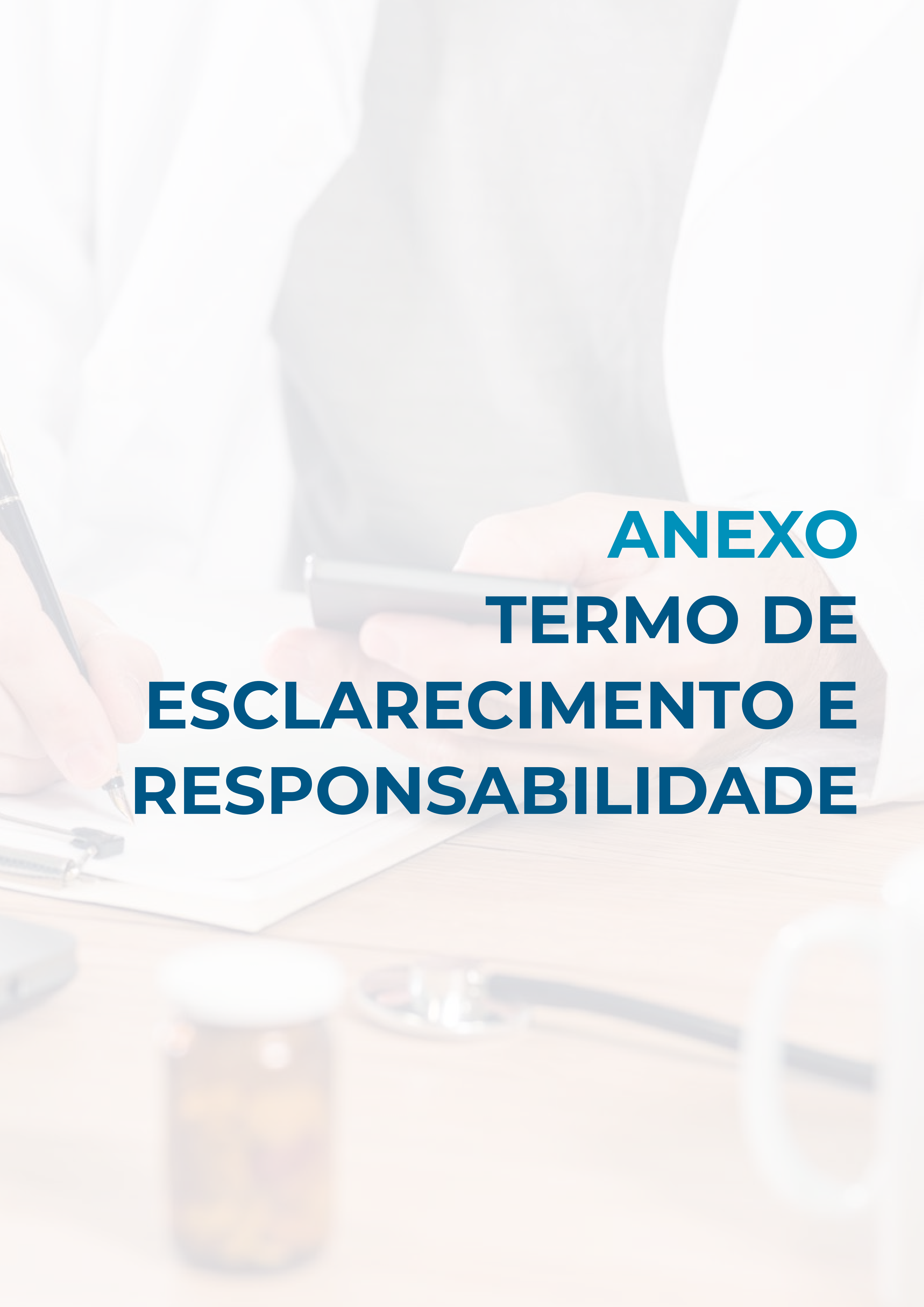

ANEXO
TERMO DE ESCLARECIMENTO E RESPONSABILIDADE

TERMO DE ESCLARECIMENTO E RESPONSABILIDADE
ÁCIDO URSODESOXICÓLICO

Eu,____(nome do(a) paciente), declaro ter sido informado(a) claramente sobre benefícios, riscos, contraindicações e principais efeitos adversos relacionados ao uso do ácido ursodesoxicólico, indicadas para o tratamento de **colangite biliar primária**.

Os termos médicos foram explicados e todas as dúvidas foram esclarecidas pelo médico ____(nome do médico que prescreve).

Assim, declaro que fui claramente informado(a) de que o medicamento que passo a receber pode trazer os seguintes benefícios:

- melhora dos sintomas e redução das complicações.

Fui também claramente informado(a) a respeito das seguintes contraindicações, potenciais efeitos adversos e riscos:

Não há dados quanto ao uso de ácido ursodesoxicólico, particularmente em mulheres grávidas. Experimentos com animais demonstraram toxicidade reprodutiva durante os estágios iniciais da gravidez.

Os riscos do uso de ácido ursodesoxicólico para o bebê durante a gestação são improváveis.

Entretanto, caso engravide, deve avisar imediatamente o médico;

- **efeitos adversos mais comuns da ácido ursodesoxicólico**: Ganho de peso, diarreia e fezes pastosas.
- **efeitos adversos mais raros** (ocorre em menos de 0,01% dos pacientes que utilizam este medicamento): intensa dor abdominal superior direita durante tratamento de colangite biliar primária; descompensação hepática foi observada em terapia de estágios avançados de cirrose biliar primária que regrediu parcialmente após a suspensão do tratamento; urticária; calcificação de cálculos.

Estou ciente de que este medicamento somente pode ser utilizado por mim, comprometendo-me a devolvê-lo caso não queira ou não possa utilizá-lo ou se o tratamento for interrompido. Sei também que continuarei a ser atendido(a), inclusive em caso de desistir de usar o medicamento.

Autorizo o Ministério da Saúde e as Secretarias de Saúde a fazerem uso de informações relativas ao meu tratamento, desde que assegurado o anonimato. ( ) Sim ( ) Não

Local:	Data:
Nome do paciente:	
Cartão Nacional do DE Saúde:	
Nome do responsável legal:	
Documento de identificação do responsável legal:	
Assinatura do paciente ou do responsável legal	
Médico responsável:<br>CRM:	 RS:
______________________________<br>Assinatura e carimbo do médico<br>Data:	

Nota: Verificar na Relação Nacional de Medicamentos Essenciais (RENAME) vigente em qual componente da Assistência Farmacêutica se encontra o medicamento preconizado neste Protocolo.

ISBN 978-85-334-2801-0

BIBLIOTECA VIRTUAL EM SAÚDE DO MINISTÉRIO DA SAÚDE
WWW.SAUDE.GOV.BR/BVS

DISQUE SAÚDE 136

SUS

MINISTÉRIO DA SAÚDE

Governo Federal
