# Supplementary material for: How to improve the dissemination of clinical practice guidelines in the Brazilian Unified Health System? Report of a pilot project
Source: Health Res Policy Syst. 2023 Mar 23;21:22. doi: 10.1186/s12961-023-00966-y (PMC10035473; doi:10.1186/s12961-023-00966-y)
Supplement: Supplementary file 4 — Additional file 4. Example of a CPG, short version. [file 12961_2023_966_MOESM4_ESM.pdf]

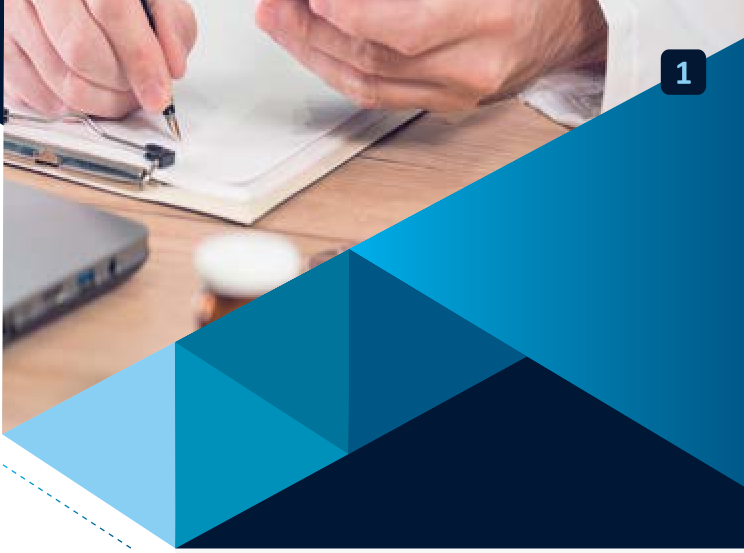

MINISTÉRIO DA SAÚDE

COLANGITE BILIAR PRIMARIA

PORTARIA CONJUNTA SCTIE/SAES/MS
Nº 11 DE 09 DE SETEMBRO DE 2019

INTRODUÇÃO

A colangite biliar primária (CBP) é uma doença hepática colestática crônica progressiva, autoimune, com reatividade sorológica para anticorpos antimitocondriais (AAM) e anticorpo antinúcleo (AAN). Caracterizada pela inflamação e destruição progressiva dos ductos biliares interlobulares de pequeno e médio calibre. Pode evoluir para cirrose, doença hepática terminal e óbito. Afeta predominantemente mulheres, na proporção de 10:1.

DIAGNÓSTICO

DIAGNÓSTICO CLÍNICO

- Mulher de meia idade que pode apresentar quadro de fadiga e prurido, dor abdominal no quadrante superior direito, anorexia, hiperpigmentação, xantelasma, hepatoesplenomegalia, icterícia, alteração no padrão do sono, osteoporose e síndrome de má-absorção
- Anormalidades colestáticas persistentes, por mais de 6 meses, em testes bioquímicos séricos hepáticos
- Correlação com outras doenças auto imunes

DIAGNÓSTICO LABORATORIAL

Confirmação diagnóstica pela presença de 2 dos 3 dos seguintes critérios (ver fluxograma):

- Elevação de enzimas colestáticas, especialmente da FA
- Presença de anticorpos antimitocondriais (AAM) ou anticorpo antinúcleo (AAN) por imunoflourescencia indireta, padrões nucleares tipo membrana nuclear e nuclear pontilhado com pontos isolados ou por Elisa, anti- gp210 e anti-sp-1001,17
- Evidência histológica de colangite destrutiva não-supurativa

DIAGNÓSTICO POR IMAGEM

- É mandatório em pacientes com evidência bioquímica de colestase.
- Colangiorressonância (preferível) ou colangiopancreatografia.

MÉTODOS HISTOLÓGICOS

A biopsia hepática é recomendada para diagnóstico de CBP em pacientes com AAM e AAN negativos, sem alterações ao exame de imagem mas com quadro clínico sugestivo.

CID-10

K74.3 Cirrose biliar primária (Colangite destrutiva não-supurativa crônica)

CRITÉRIOS DE INCLUSÃO

Pacientes de ambos os sexos, que preencham pelo menos, 2 de 3 dos seguintes critérios diagnósticos:

- Elevação de enzimas colestáticas, especialmente da FA
- Presença de AAM ou AAN (padrões específicos à imunofluorescência ou anti-gp210 e anti-sp-100 por Elisa)
- Evidência histológica de colangite destrutiva não-supurativa

CRITÉRIOS DE EXCLUSÃO

- Alergia a ácido ursodesoxicólico ou a qualquer um dos componentes da formulação
- Úlcera péptica (gástrica ou duodenal) em fase ativa
- Doença intestinal inflamatória e outras condições do intestino delgado, cólon e fígado, que possam interferir com a circulação entero-hepática dos sais biliares (ressecção ileal e estoma, colestase intra e extra-hepática, doença hepática grave)
- Cólicas biliares frequentes
- Inflamação aguda da vesícula biliar ou trato biliar
- Oclusão do trato biliar (oclusão do ducto biliar comum ou um ducto cístico)
- Contratilidade comprometida da vesícula biliar
- Cálculos biliares calcificados radiopacos
- Pacientes com intolerância a galactose, deficiência de Lapp lactase ou má absorção de glicose galactose

FLUXOGRAMA DE DIAGNÓSTICO DE COLANGITE BILIAR PRIMÁRIA (CBP)

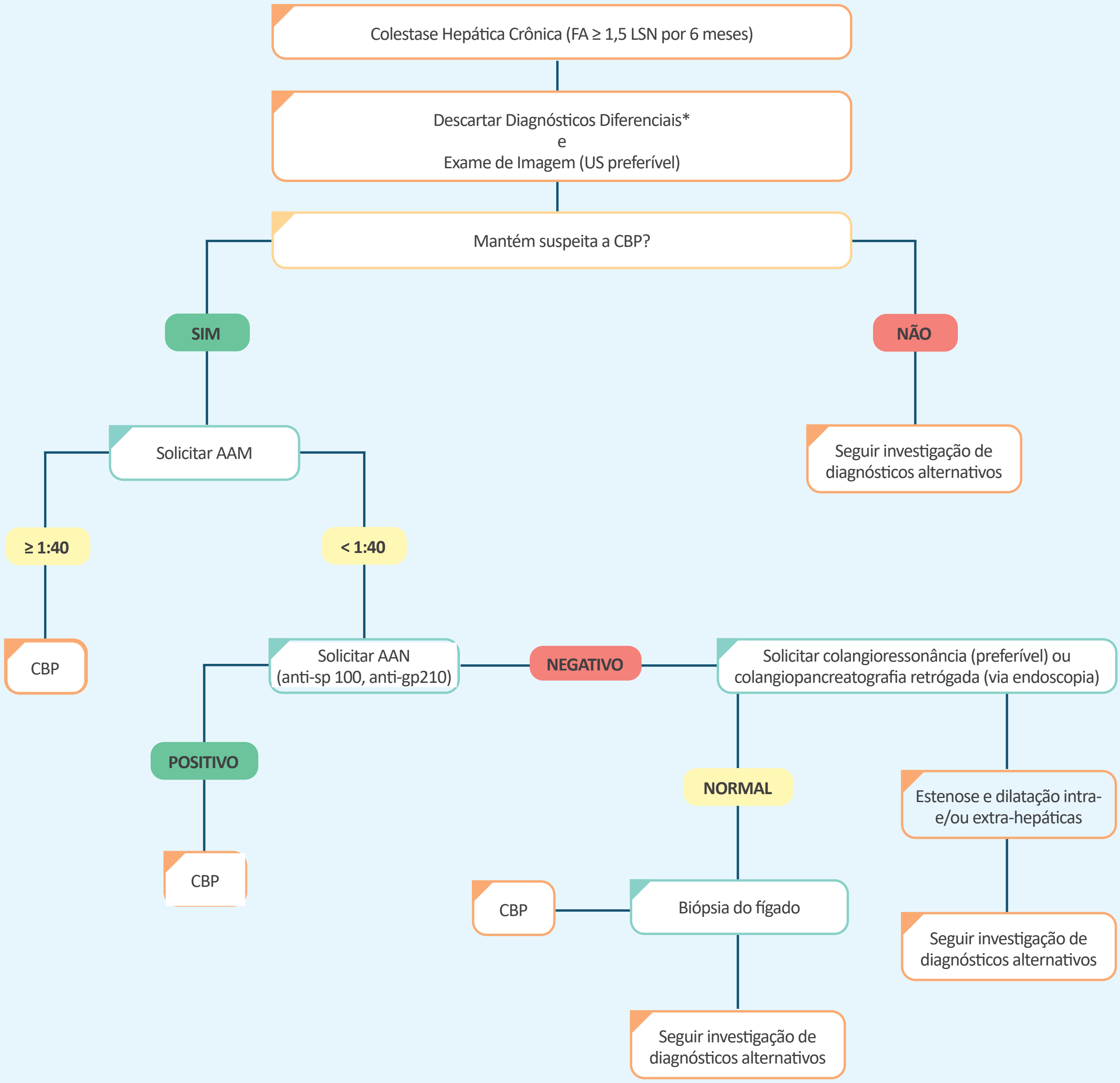

* Diagnósticos especiais:
Colestase medicamentosa,
Hepatites virais,
Obstrução biliar,
Sarcoidose,
Hepatite autoimune,
Colangite esclerosante primária.

Legenda: FA, fosfatase alcalina; LSN, limite superior da normalidade; AAM, anticorpo antimitocôndria; AAN, anticorpo antinúcleo; CBP, colangite biliar primária.

TRATAMENTO

Benefícios esperados: redução dos sintomas de prurido e fadiga, reversão ou regressão das lesões hepáticas, aumento na sobrevida livre de transplante, melhora dos sintomas e da qualidade de vida dos pacientes.

Tratamento colangite biliar primaria

Tratamento medicamentoso

Ácido ursodesoxicólico
Dose: 13 mg a 15 mg/kg/dia, via oral, dose única ou dividida em até quatro doses diárias Apresentação:

Tratamento não medicamentoso

- Suspender consumo de álcool
- Cessação do tabagismo
- Evitar alimentos com cafeína
- Exercício físico regular
- Dieta balanceada

O transplante hepático deve ser considerado quando as demais intervenções disponíveis se mostrarem ineficazes e em quadros avançados.

CRITÉRIOS DE INTERRUPÇÃO DO TRATAMENTO

O tempo de tratamento não pode ser pré-determinado e deve ser realizado a longo prazo. Espera-se benefícios em termos de retardo ou interrupção da evolução da doença, quando realizado de maneira contínua.

MONITORIZAÇÃO

Após 12 meses do início do tratamento realizar testes bioquímicos hepáticos: fosfatase alcalina, bilirrubina, AST, ALT, albumina e plaquetas conforme a tabela 1.

Tabela 1 - Avaliação da resposta ao tratamento com ácido ursodesoxicólico em pacientes com CBP1

Definições binárias qualitativas	Tempo (meses)	Falha ao tratamento
Barcelona	12	Redução < 40% na ALP e ALP ≥ 1 x LSN
Paris I	12	ALP ≥ 3 x LSN ou AST ≥2 x LSN ou bilirrubina >1mg/dl
Roterdã	12	Bilirrubina ≥1 x LSN ou albumina <1 x LSN
Toronto	24	ALP >1,67 x LSN
Paris II	12	ALP≥1,5 x LSN ou AST≥1,5 x LSN ou bilirrubina > 1mg/dl
**Sistema de escores contínuos**	**Tempo (meses)**	**Parâmetros de pontuação**
UK-CBP	12	Bilirrubina, ALP e AST (ou ALT) em 12 meses. Albumina e contagem de plaquetas no baseline
GLOBE	12	Bilirrubina, ALP, albumina, contagem de plaquetas em 12 meses. Idade no baseline.

Legenda: GGT: gama-glutamiltransferase; ALP: fosfatase alcalina; AST/TGO: aspartato aminotranferase; LSN: limite superior do normal.

As informações inseridas neste material tem a finalidade de direcionar a consulta rápida dos principais temas abordados no PCDT. A versão completa corresponde a Portaria Conjunta nº 11 de 09 de setembro de 2019 e pode ser acessada em http://conitec.gov.br/index.php/protocolos-e-diretrizes.

DISQUE SAÚDE 136
SUS MINISTÉRIO DA SAÚDE Governo Federal
